# Development of STS and CAPS markers for variety identification and genetic diversity analysis of tea germplasm in Taiwan

**DOI:** 10.1186/1999-3110-55-12

**Published:** 2014-02-01

**Authors:** Chih-Yi Hu, You-Zen Tsai, Shun-Fu Lin

**Affiliations:** 1 Department of Agronomy, National Taiwan University, Taipei, 106 Taiwan; 2 Wunshan Branch, Tea Research and Extension Station, Council of Agriculture, Executive Yuan, New Taipei City, 231 Taiwan; 3 Tea Research and Extension Station, Council of Agriculture, Executive Yuan, Taoyuan, 324 Taiwan

**Keywords:** Tea plant, *Camellia sinensis*, *Camellia formosensis*, CAPS markers, STS markers, Variety identification, Genetic diversity analysis

## Abstract

**Background:**

Tea (*Camellia sinensis*) is an important economic crop in Taiwan. Particularly, two major commercial types of tea (Paochong tea and Oolong tea) which are produced in Taiwan are famous around the world, and they must be manufactured with specific cultivars. Nevertheless, many elite cultivars have been illegally introduced to foreign countries. Because of the lower cost, large amount of “Taiwan-type tea” are produced and imported to Taiwan, causing a dramatic damage in the tea industry. It is very urgent to develop the stable, fast and reliable DNA markers for fingerprinting tea cultivars in Taiwan and protecting intellectual property rights for breeders. Furthermore, genetic diversity and phylogenetic relationship evaluations of tea germplasm in Taiwan are imperative for parental selection in the cross-breeding program and avoidance of genetic vulnerability.

**Results:**

Two STS and 37 CAPS markers derived from cytoplasmic genome and ESTs of tea have been developed in this study providing a useful tool for distinguishing all investigated germplasm. For identifying 12 prevailing tea cultivars in Taiwan, five core markers, including each one of mitochondria and chloroplast, and three nuclear markers, were developed. Based on principal coordinate analysis and cluster analysis, 55 tea germplasm in Taiwan were divided into three groups: *sinensis* type (*C. sinensis* var. *sinensis*), *assamica* type (*C. sinensis* var. *assamica*) and Taiwan wild species (*C. formosensis*). The result of genetic diversity analysis revealed that both *sinensis* (0.44) and *assamica* (0.41) types had higher genetic diversity than wild species (0.25). The close genetic distance between the first (Chin-Shin-Oolong) and the third (Shy-Jih-Chuen) prevailing cultivars was found, and many recently released varieties are the descents of Chin-Shin-Oolong. This implies the potential risk of genetic vulnerability for tea cultivation in Taiwan.

**Conclusions:**

We have successfully developed a tool for tea germplasm discrimination and genetic diversity analysis, as well as a set of core markers for effective identification of prevailing cultivars in Taiwan. According to the results of phylogenetic analysis on prevailing tea cultivars, it is necessary to broaden genetic diversity from wild species or plant introduction in future breeding programs.

**Electronic supplementary material:**

The online version of this article (doi:10.1186/1999-3110-55-12) contains supplementary material, which is available to authorized users.

## Background

Tea (*Camellia sinensis*) is one of the most important beverage crops around the world and also a significant economic crop in Taiwan. Currently, there are 14,091 hectares of tea farms in Taiwan, producing 17,310 tons per year (Council of Agriculture 2012). Tea has been planted in Taiwan since 200 years ago, and has been manufactured into different types of tea in accordance with different eras and production areas (Chiu 1988; Jun and Lin 1997). Because different types of tea are produced with specific cultivars, numerous tea cultivars are grown in Taiwan. Paochong tea and Oolong tea are two major types of tea currently produced in Taiwan, whereas black and green tea are considered to be minor types. The cultivars suitable for making Paochong or Oolong tea are cultivars Chin-Shin-Oolong, TTES-12, Shy-Jih-Chuen, Chin-Shin-Dahpan, and TTES-13. Whereas cultivar Chin-Shin-Gantzy is fitting for green tea, and TTES-8 and TTES-18 are suitable for black tea (Tsai et al. 2004b). Furthermore, there are many other germplasm including landraces, introduced varieties, and wild species that could be selected or utilized for breeding new varieties.

Tea is a woody, perennial, and out-crossing crop that is highly heterozygous (Barua 1963). In tea breeding, the key points for parental selection are superior traits from parents and their wide-ranging genetic diversity that prevent the weakness of progenies (Bandyopadhyay 2011). Furthermore, many elite cultivars developed in Taiwan have been illegally introduced to China, Vietnam, Thailand, Indonesia, and so on. Because of the lower cost, large number of “Taiwan-type tea” are produced and imported to Taiwan, causing a dramatic damage in the tea industry. Therefore, seedlings and products of tea have been protected by the “Plant Variety and Plant Seed Act” which was enacted in 2004. In addition, the scientific database for identifying and examining varieties of tea should be well developed for the suspicious torts.

The simple method for genetic diversity assessment and variety identification of tea or its commercial product (processed tea) is based on the morphological traits. However, the available morphological traits are limited in number and easily affected by environments and growth stages of tea (Gunasekare 2007; Bandyopadhyay 2011). DNA markers are genetic markers that came from various classes of DNA mutations and rearrangements (Collard et al. 2005). Compared with morphological traits, DNA markers have numerous advantages such as multiple marker types, relative abundance of polymorphism, extensive genomic coverage, not disturbed by growth stage and tissue of plants, not affected by environment and gene expression, only a small quantity of DNA needed for assay, only a short period required for analyzing large amounts of samples, and more reproducible (Powell et al. 1996; Collard et al. 2005; Jones et al. 2009). DNA markers, including RAPD (randomly amplified polymorphic DNA), ISSR (inter-simple sequence repeat) and AFLP (amplified fragment length polymorphism) have been well developed for genetic fingerprinting and phylogenetic studies of tea in Taiwan (Lai et al. 2001; Tsai et al. 2003; Hu et al. 2005; Lin et al. 2005). Nevertheless, these markers are dominant, and their reproducibility and capacity for variety identification are less than targeted and locus-specific DNA markers, such as STS and CAPS.

STS (ssequence tagged site) is a relatively short and single-copy DNA sequence that can be specifically amplified by PCR (Olson et al. 1989). CAPS (cleaved amplified polymorphic sequence) or PCR-RFLP (polymerase chain reaction **-** restriction fragment length polymorphism) utilizes amplified DNA fragments digested with a restriction endonuclease to display restriction site polymorphisms (Konieczny and Ausubel 1993). STS and CAPS markers are co-dominant, locus-specific, and more reproducible. They have various advantages including their genotypes which are easily scored and interpreted, and only a small quantity of DNA is needed for one assay. Also the cleaved and un-cleaved amplification products can be adjusted arbitrarily by the appropriate placement of the PCR primers. The procedure is technically simple with robust results because the amplification product is always obtained (Drenkard et al. 1997).

DNA markers could be developed from whole nuclear genome or expressed sequence tags (ESTs). Because the whole genome sequences of tea plant are not available and updated, it is feasible to develop nuclear markers from ESTs database. ESTs are short cDNA sequences reversely transcribed from mRNA. In general, by using EST-derived primer pairs to amplify nuclear genome, the amplicons may consist of intron sequences that displayed higher variation to develop informative markers for variety identification (Shu et al. 2010). Besides, DNA markers could be also derived from the cytoplasmic genome, such as the mitochondria genome (mtDNA) and chloroplast genome (cpDNA). The cytoplasmic CAPS markers are not only maternal inherited from haploid genome (Kaundun and Matsumoto 2011), but also have a slower nucleotide substitution rate than the nuclear DNA (Palmer 1992). Because of conservative evolution, they have been widely used in detecting geographical origins of plant species (Kaundun and Matsumoto 2002; Katoh et al. 2003) and population differentiation (Schaal and Olsen 2000).

The aim of this study is to develop a stable, fast and reliable STS and CAPS DNA markers for fingerprinting commercial tea varieties in Taiwan and protect intellectual property rights for breeders. Furthermore, genetic diversity and phylogenetic relationship of tea germplasm in Taiwan are assessed to provide information for parental selection.

## Methods

### Plant materials and DNA extraction

A total of 55 germplasm were analyzed in this study, including 22 selected from crossing between varieties, nine local cultivars (landraces), 16 introduced varieties, and eight wild species. According to taxonomy, 22 *C. sinensis* var. *sinensis* (S), 12 *C. sinensis* var. *assamica* (A), 11 *C. sinensis* var. *sinensis* × var. *assamica* (SA), two *C. sinensis* var. *assamica* × var. *assamica* (AS), seven *C. formosensis* (F), and one *C. formosensis* var. *yungkangensis* (FY) are classified (Hu et al*.*^2005^; Su 2007; Su et al. 2009). Except four (I4 ~ I7) samples were obtained from the tea garden of Tung Pang Black Tea CO. LTD. in Nantou County, Taiwan. All samples were collected from the germplasm garden at the Tea Research and Extension Station in Taoyuan County, Taiwan (Table 1).

**Table 1:** **List of the 55 tea germplasm used in this study**

Code	Variety/Line	Species/Variety^§^	Type of germplasm	Processing suitability^#^	Origin or Parent^@^
H1	TTES No.1 (TTES-1)	SA	Developed variety	B,G,O	Chin-Shin-Dahpan (TW) × Kyang (IN)
H2	TTES No.2 (TTES-2)	SA	Developed variety	B,G,O	Dah-Yeh-Oolong (TW) × Jaipuri (IN)
H3	TTES No.3 (TTES-3)	SA	Developed variety	B,G	Horng-Shin-Dahpan (TW) × Manipuri (IN)
H4	TTES No.4 (TTES-4)	SA	Developed variety	B, G	Horng-Shin-Dahpan (TW) × Manipuri (IN)
H5	TTES No.5 (TTES-5)	S	Developed variety	O, P, G	Fwu-Jou line (CN)
H6	TTES No.6 (TTES-6)	S	Developed variety	G, B, O	Chin-Shin-Oolong line (TW)
H7	TTES No.7 (TTES-7)	A	Developed variety	B	Shan line (TH)
H8	TTES No.8 (TTES-8)	A	Developed variety	B	Jaipuri line (IN)
H9	TTES No.9 (TTES-9)	SA	Developed variety	G,B	Horng-Shin-Dahpan (TW) × Kyang (IN)
H10	TTES No.10 (TTES-10)	SA	Developed variety	G,B	Hwang-Gan (TW) × Jaipuri (IN)
H11	TTES No.11 (TTES-11)	SA	Developed variety	G,B	Dah-Yeh-Oolong (TW) × Jaipuri (IN)
H12	TTES No.12 (TTES-12)	S	Developed variety	O, P	Tainon-8^*^ (TW) × Ying-Jy-Horng-Shin (TW)
H13	TTES No.13 (TTES-13)	S	Developed variety	O, P	Ying-Jy-Horng-Shin (TW) × Tainon-80^*^ (TW)
H14	TTES No.14 (TTES-14)	SA	Developed variety	O, P	Tainon-983^*^ (TW) × Bair-Mau-Hour (TW)
H15	TTES No.15 (TTES-15)	SA	Developed variety	O, G	Tainon-983^*^ (TW) × Bair-Mau-Hour (TW)
H16	TTES No.16 (TTES-16)	SA	Developed variety	G, P	Tainon-355^*^ (TW) × Tainon-1958 (TW)
H17	TTES No.17 (TTES-17)	SA	Developed variety	O, G	Tainon-355^*^ (TW) × Tainon-1958 (TW)
H18	TTES No.18 (TTES-18)	A	Developed variety	B	Burma (MM) × Taiwanese wild tea (TW)
H19	TTES No.19 (TTES-19)	S	Developed variety	O, P	TTES-12 (TW) × Chin-Shin-Oolong (TW)
H20	TTES No.20 (TTES-20)	S	Developed variety	O, P	2022^*^ (TW) × Chin-Shin-Oolong (TW)
H21	TTES No.21 (TTES-21)	AS	Developed variety	B	FKK-1 line
H22	FKK-1	AS	Developed variety	B	Kyang (IN) × Kimen (CN)
L1	Chin-Shin-Oolong	S	Landraces	O, P	Planting around Taiwan
L2	Shy-Jih-Chuen	S	Landraces	O, P	Planting in Nantou, Taiwan
L3	Chin-Shin-Dahpan	S	Landraces	O, P, G, B	Planting in north-west of Taiwan
L4	Chin-Shin-Gantzy	S	Landraces	G	Planting in New Taipei City, Taiwan
L5	Ying-Jy-Horng-Shin	S	Landraces	O	Planting in New Taipei City, Taiwan
L6	Dah-Yeh-Oolong	S	Landraces	O, G	Planting in north and east of Taiwan
L7	Hwang-Gan	S	Landraces	B	Planting in north-west of Taiwan
L8	Bair-Mau-Hour	S	Landraces	O	Planting in north of Taiwan
L9	Horng-Shin-Dahpan	S	Landraces	G	Planting in north-west of Taiwan
I1	Kimen	S	Introduced variety	B	Original from China
I2	Burma	A	Introduced variety	B	Original from Myanmar
I3	Shan	A	Introduced variety	B	Original from Thailand
I4	Shan-1	A	Introduced variety	B	Original from Thailand
I5	Shan-2	A	Introduced variety	B	Original from Thailand
I6	Shan-3	A	Introduced variety	B	Original from Thailand
I7	Shan-4	A	Introduced variety	B	Original from Thailand
I8	Manipuri	A	Introduced variety	B	Original from India
I9	Jaipuri	A	Introduced variety	B	Original from India
I10	Kyang	A	Introduced variety	B	Original from India
I11	Tiee-Guan-In	S	Introduced variety	O, P	Original from China
I12	Wuu-Yi	S	Introduced variety	O, P	Original from China
I13	Shoei-Shian	S	Introduced variety	O, P	Original from China
I14	Shiang-Yuan	S	Introduced variety	O, P	Original from China
I15	Hann-Koou	S	Introduced variety	B	Original from China
I16	Fwu-Jou	S	Introduced variety	P	Original from China
W1	De-Hua-She wild tea	F	wild tea	B	Original from Nantou, Taiwan
W2	Fong-Huang wild tea	F	wild tea	B	Original from Nantou, Taiwan
W3	Mei-Yuan wild tea	F	wild tea	B	Original from Nantou, Taiwan
W4	Le-Ye wild tea	F	wild tea	B	Original from Chiayi, Taiwan
W5	Ming-Hai wild tea	F	wild tea	B, O	Original from Kaohsiung, Taiwan
W6	Nan-Fong wild tea	F	wild tea	B, O	Original from Kaohsiung, Taiwan
W7	Long-Tou wild tea	F	wild tea	B, O	Original from Kaohsiung, Taiwan
W8	Yung-Kang wild tea	FY	wild tea	B, G	Original from Taitung, Taiwan

Note^§^: Based on Hu et al. (2005), Su (2007), and Su et al. (2009).

Abbreviation *S*:*C. sinensis* var. *sinensis*, *A*: *C. sinensis* var. *assamica*, *SA*: *C. sinensis* var. *sinensis* × var. *assamica* hybrid, *AS*: *C. sinensis* var. *assamica* × var. *assamica* hybrid, *F*: *C. formosensis*, *FY*: *C. formosensis* var. *yungkangensis*.

Note^#^: *G* green tea, *P* Paochong tea, *O* oolong tea, *B* black tea.

Note^@^: TW *-* Taiwan, IN *-* India, CN *-* China, TH *-* Thailand, MM *-* Myanmar.

Note*: Tainon-983: Hwang-Gan × Kyang; Tainon-335: Dah-Yeh-Oolong × Kyang;

Tainon-1958: Tainon-20 × Bair-Mau-Hour; Tainon-8: Hwang-Gan × Chin-Shin-Oolong;

2022: Dah-Yeh-Oolong × Tainon-20; Tainon-20: Hann-Koou line; Tainon-80: Hann-Koou line.

The DNA was isolated from buds and leaves by using a modification of Doyle and Doyle (1990) described by Hu et al. (2005).

### Design of STS markers

To develop cytoplasmic STS markers, primer pairs of two chloroplast and seven mitochondria were designed according to Hu (2004). In addition, 54 primer pairs of nuclear STSs, including four developed by Kaundun and Matsumoto (2003,2004), and 50 based on the public EST database (dbEST) of NCBI (National Center for Biotechnology Information, USA, http://www.ncbi.nlm.nih.gov/) were designed, using the Primer3 software (Rozen and Skaletsky 2000) (http://frodo.wi.mit.edu/). These primer pairs were prescreened with eight cultivars comprising of the following: TTES-8; TTES-12; TTES-13; TTES-18; TTES-19; TTES-20; Chin-Shin-Oolong and Shy-Jih-Chuen. The amplification was performed in a total volume of 38 μL containing 80 ng genomic DNA, 0.3 μM each primer, 4.7 mM MgCl_2_, 0.27 mM dNTPs, and 1 U of *Taq* DNA polymerase (Invitrogen by Life Technologies). The amplification was done by T-Gradient (Biometra, Germany) with denaturation at 94°C for 4 min; 40 cycles of 94°C for 30 s, 55-60°C (depending on the primer pair) for 30 s, and 72°C for 30 s; and final extension at 72°C for 4 min.

### Design of CAPS markers

Nuclear amplicons that amplified two bands with length polymorphisms were directly applied as STS markers. Meanwhile, the DNA bands were sequenced by ABI PRISM 3730 DNA Analyzer (Applied Biosystems, USA) once the PCR products were less than 1 kb. For SNPs (single nucleotide polymorphism) and InDels (insertion/deletion) screening, sequence analyses were conducted with SeqMan Pro v.7.1 software (DNAStar, Inc., Madison, WI, USA). The sequences with SNPs or InDels were converted to CAPS markers by SNP2CAPS software (Thiel et al. 2004). To check restriction patterns, PCR reactions were performed in a final volume of 11.7 μL with 1× *Taq* buffer, 2 mM MgCl_2_, 0.27 mM dNTPs, 0.26 μM each primer, 1 U *Taq* DNA polymerase (Invitrogen by Life Technologies), and 40 ng DNA. Amplification was done by T-Gradient (Biometra, Germany) with programmed for 5 min preheating at 94°C followed by 35 cycles of 30 s at 94°C, 30 s at 55-60°C (depending on the primer pair) and 1 min at 72°C for the denaturation, annealing and extension steps, respectively. There was a final incubation for 10 min at 72°C. Amplification products were analyzed on 2% agarose gels stained with ethidium bromide to check the fragments being amplified. Amplified fragments were digested with restriction enzymes to detect CAPS and the products were resolved by electrophoresis on 2% agarose gels.

### Data analysis and variety identification

The haploid and diploid types for cytoplasmic and nuclear markers were respectively scored, and each allele was assigned an alphabet for a particular primer set/enzyme combination. The polymorphism information of STS and CAPS markers was analyzed by PowerMarker v.3.25 (Liu and Muse 2005) to investigate the number of alleles and polymorphism information content (PIC) per marker.

$$\mathrm{PIC}=1-\left(\sum_{i=1}^{n}Pi^{2}\right)-\sum_{i=1}^{n-1}\sum_{j=i+1}^{n}2Pi^{2}Pj^{2}$$

, where *Pi* and *Pj* are the frequencies of the *i* th and *j* th alleles, and *n* is the number of alleles (Botstein et al. 1980).

Based on the identified STS and CAPS markers, the core markers and the flow chart for identifying 12 prevailing cultivars in Taiwan, including Chin-Shin-Oolong, TTES-12, Shy-Jih-Chuen, Chin-Shin-Dahpan, TTES-13, Chin-Shin-Gantzy, TTES-8, TTES-18, TTES-7, TTES-19, TTES-20, and TTES-21, were developed.

### Genetic diversity analysis

In this study, the tea germplasm consist of three main groups, including *sinensis* type (S and SA), *assamica* type (A and AS) and wild species in Taiwan (F and FY) (shown in Table 1). The genetic diversity of those germplasm was analyzed by Popgene v.1.32 (Yeh and Boyle 1997) to estimate the observed number of alleles (N_A_), the effective number of alleles (N_e_), the observed heterozygosity (H_O_), the Nei’s gene diversity (H), and Shannon’s Information index (I) per group.

### Cluster analysis and principle coordinates analysis

Both the analyses of average genetic distances among three main groups and genetic distances between the pairs of germplasm were based on modified Roger’s distance (MRD) method (Wright 1978) by using TFPGA v.1.3 (Miller 1997). Upon the genetic distances between all pairwise combinations MRD, the cluster analysis and principal coordinate analysis (PCoA) were completed with NTSYSpc v.2.10 (Rohlf 1997). A dendrogram of the genetic relationships was developed by unweighted pair group method with arithmetic mean algorithm (UPGMA) using cluster analysis. The principal coordinate analysis (PCoA) was performed and the first two extracted coordinates extracted were used to derive the PCoA plot.

## Results

### Polymorphism of STS and CAPS markers

The STS and CAPS markers in this study were derived from cytoplasmic genome and nuclear ESTs. From six polymorphic DNA sequences of cytoplasmic genome, 14 SNPs and same amount of InDels were screened and successfully designed for three chloroplast CAPS (C01 ~ C03) and seven mitochondria CAPS (M01 ~ M07) markers. A total of 54 nuclear EST primer pairs, including four pairs from the previous study (Kaundun and Matsumoto 2003,2004) and 50 pairs designed from public EST database of NCBI, as well as 27 primer pairs which amplified the expected size of amplicons. However, the remaining 27 primer pairs did not yield any scorable amplicon or yielded amplicons longer than 1 kb. In the expected size of 27 amplicons, 11 had no SNP, three had SNP (but without the restriction site), and the remaining 13 amplicons had 90 SNPs. Meanwhile, the four InDels could be successfully transferred into two STS (PAL and F3H) and 27 CAPS markers (including G01 ~ G27). For example, one SNP of an EST sequence coding zinc finger protein was designed for CAPS marker shown in Additional file 1: Figure S1. The detailed information of the two STS and 37 CAPS markers (including 10 cytoplasmic markers and 27 nuclear markers) are listed in Table 2.

**Table 2:** **Primer sequences and restriction enzymes of STS and CAPS markers yielding polymorphic bands for tea germplasm**

Marker*	Forward primer	Reverse primer	Size (bp)	Annealing temp.	Nuclease	Size of major bands (bp)	Allele number	PIC^§^	Reference sequence^※^	Prediction function
C01	GAGGGGAAGGATGGATTGTT	GTGCCACAAATGACCTACGA	677	55°C	*Taq* I	A: 677 B: 552 + 125	2	0.15	AY741470	photosystem II CP43 protein
C02	GAGGGGAAGGATGGATTGTT	GTGCCACAAATGACCTACGA	677	55°C	*Bsr* DI	A: 677 B: 483 + 194	2	0.13	AY741470	photosystem II CP43 protein
C03	GAGAGAGAGGGATTCGAACC	GTTTTTGGAGCTGGGATGAA	659	55°C	*Sfa* NI	A: 659 B: 449 + 210	2	0.27	AY839880	*trnS ~ trnfM*
M01	TGGTGAGGAGCATTGTTTTG	GAGCAAACACTCGAACGTGA	836	55°C	*Eco* RI	A: 836 B: 509 + 327	2	0.15	AY839898	NADH dehydrogenase subunit 1
M02	CCAATTTTTGGGCCAATTCC	TCTCTAAAGGGGCGTAAGCA	610	55°C	*Hinc* II	A: 610 B:385 + 225	2	0.31	AY845285	NADH dehydrogenase subunit 5
M03	GCCGGAAAAATAACAGACGA	AAAAGGAAGGTTGGGTGCTT	458	55°C	*Bbs* I	A: 458 B: 308 + 149	2	0.28	AY845314	NADH dehydrogenase subunit 7
M04	GATAGGAGCATTCGGTGGAA	CGGTAACCAAAGCGTATCGT	844	55°C	*Hph* I	A: 805 + 35 B: 492 + 313 + 35	2	0.28	AY845314	NADH dehydrogenase subunit 7
M05	ACAGCACCTTTTTCCCCTCT	CATAACACGGCTCTCCCACT	686	55°C	*Xmn* I	A: 686 B:443 + 243	2	0.34	AY845314	NADH dehydrogenase subunit 7
M06	TGAATGAATCCCATCCCCTA	GGCATACAACCGAAACGACT	413	55°C	*Rsa* I	A: 413 B:309 + 104	2	0.35	AY845285	NADH dehydrogenase subunit 5
M07	TAGCTATGCCCTGCTTGGTC	CCTGTCTGTCGTACCGTTGA	667	55°C	*Bss* SI	A: 667 B: 460 + 207	2	0.28	AY845298	NADH dehydrogenase subunit 7
G01	TGCTTTGCGTCAATAACTGC	TGATACATCCTCGCCAACAA	647	55°C	*Hph* I	A:647 B:441 + 206	2	0.25	DQ869863	zinc finger protein
G02	GCCTATCTAATCTACTCGGCTTTCT	AGTAACACTAACCCACCCAACAATA	834	55°C	*Fsp* I	A:834 B:645 + 189	2	0.36	AB117640	ammonium transporter
G03	GCCTATCTAATCTACTCGGCTTTCT	AGTAACACTAACCCACCCAACAATA	834	55°C	*Ava* I	A:834 B:729 + 105 C:501 + 228 + 105	3	0.55	AB117640	ammonium transporter
G04	GAGACAGAGGACTACTTCGATTCAG	GAATCAGAAATGATACAGAGGAGGA	721	55°C	*Mse* I	A:721 B:447 + 274	2	0.37	AB247282	cyclin D3-1
G05	GAGACAGAGGACTACTTCGATTCAG	GAATCAGAAATGATACAGAGGAGGA	721	55°C	*Rsa* I	A:721 B:571 + 150	2	0.30	AB247282	cyclin D3-1
G06	GCCTATCTAATCTACTCGGCTTTCT	AGTAACACTAACCCACCCAACAATA	834	55°C	*Nde* I	A:834 B:749 + 85	2	0.25	AB117640	ammonium transporter
G07	GCCTATCTAATCTACTCGGCTTTCT	AGTAACACTAACCCACCCAACAATA	834	55°C	*Hph* I	A:510 + 147 B:468 + 147 + 120 + 57	2	0.31	AB117640	ammonium transporter
G08	GCCTATCTAATCTACTCGGCTTTCT	AGTAACACTAACCCACCCAACAATA	834	55°C	*Ava* II	A:443 + 190 + 134 + 67 B: 324 + 67	2	0.15	AB117640	ammonium transporter
G09	GCCTATCTAATCTACTCGGCTTTCT	AGTAACACTAACCCACCCAACAATA	834	55°C	*Bst* UI	A:575 + 204 + 55 B:455 + 204 + 120 + 55	2	0.07	AB117640	ammonium transporter
G10	TGAGACAACATTATGGTCGATAGAA	ATACTCCTTGCAAACTTCTGAATTG	750	55°C	*Bst* YI	A:750 B:590 + 160	2	0.14	AY641731	trans-cinnamate 4-hydroxylase
G11	TGAGACAACATTATGGTCGATAGAA	ATACTCCTTGCAAACTTCTGAATTG	750	55°C	*Bst* UI	A:750 B:470 + 280	2	0.10	AY641731	trans-cinnamate 4-hydroxylase
G12	ACGACTACAGCTTCTTTCTCTACCA	ATACACCTCGTCGACATACTTCTTC	824	55°C	*Ava* I	A:824 B: 527 + 297 C:325 + 202	3	0.43	AB114913	ammonium transporter
G13	CCATCAAATCCATTGGGAAC	AAGACGAGCCAGGAGAAACA	580	60°C	*Mbo* II	A:580 B:449 + 131	2	0.10	EF205149	1-aminocyclopropane-1-carboxylate synthase
G14	CCATCAAATCCATTGGGAAC	AAGACGAGCCAGGAGAAACA	580	60°C	*Bsr* DI	A:359 + 221 B:455 + 125 C:221 + 218 + 141 D:260 + 195 + 125 E:359 + 125 + 96 F:455 + 320 + 260 + 125 G:260 + 125 + 99 + 96	7	0.52	EF205149	1-aminocyclopropane-1-carboxylate synthase
G15	GCCTATCTAATCTACTCGGCTTTCT	AGTAACACTAACCCACCCAACAATA	834	55°C	*Bsa* JI	A:546 + 288 B:318 + 288 + 228 C:546 + 183 + 105 D: 318 + 228 + 183 + 105	5	0.58	AB117640	ammonium transporter
G16	CCTACAAAACAGTCATAAGCCAACT	ACGAAAACACTCTTGATCAGTAAGG	572	55°C	*Bbv* I	A:309 + 263 B:263 + 166 + 143	2	0.04	AB015047	PR-1 like protein
G17	ACGTGTGTGTTTCATTTGCC	AACCCAATGATGTGTAAGTG	556/401	55°C	*Mbo* II	A:556 B:455 + 101 C:300 + 101	3	0.28	D26596^b^	phenylalanine ammonia-lyase
G18	CTTACGGCTCTCGCAGAAGA	GAACCGTGATCCAGGTTTTG	1100/930	55°C	*Hind* III	A:1100 B:930 C:650 + 280	3	0.49	AY641730	flavanone 3-hydroxylase
G19	TGCTTTGCGTCAATAACTGC	TGATACATCCTCGCCAACAA	647	55°C	*Hpa* II	A:619 + 28 B:463 + 156 + 28	2	0.37	DQ869863	zinc finger protein
G20	ACGTGTGTGTTTCATTTGCC	AACCCAATGATGTGTAAGTG	556/401	55°C	*Hph* I	A:556 + 401 B:401 C:319 + 237 D:237	4	0.47	D26596^a^	phenylalanine ammonia-lyase
G21	ACGTGTGTGTTTCATTTGCC	AACCCAATGATGTGTAAGTG	556/401	55°C	*Taq* I	A:556 B:460 + 96 C:305 + 96	3	0.53	D26596^a^	phenylalanine ammonia-lyase
G22	CTTACGGCTCTCGCAGAAGA	GAACCGTGATCCAGGTTTTG	1100/930	55°C	*Bst* YI	A:930 B:680 + 250 C:510 + 420 D:680 + 420	4	0.62	AY641730	flavanone 3-hydroxylase
G23	CTTACGGCTCTCGCAGAAGA	GAACCGTGATCCAGGTTTTG	1100/930	55°C	*Bbs* I	A:1100 B:930 C:600 + 330	3	0.55	AY641730	flavanone 3-hydroxylase
G24	CCAGGAACACCAACAACCCGT	CCATGCTGCTTTCTCTGCCAA	958	55°C	*Hind* III	A:958 B:550 + 408	2	0.35	AB018685^b^	dihydroflavonol 4-reductase
G25	GATCCTTCAGACATGCAGAGC	CACTTCCTCAAGTGATGCAAA	960	55°C	*Rsa* I	A:960 B:605 + 355	2	0.21	EF526217	caffeine synthase
G26	CGACCATCCTGCAATTTTCT	AACGTCATTAGGACCTTCAATCG	299	55°C	*Taq* I	A:310 B:299 C:290 D:277	4	0.49	EF205148	leucoanthocyanidin reductase
G27	GGTGCTCAGGACATGGTTTT	CGCTCTATTCCCTGCAAGTC	377	55°C	*Hph* I	A:377 B:198 + 179	2	0.37	DQ194358	flavonoid 3′,5′-hydroxylase
PAL	ACGTGTGTGTTTCATTTGCC	AACCCAATGATGTGTAAGTG	556/401	55°C	-	A:556 B:401	2	0.27	D26596^a^	phenylalanine ammonia-lyase
F3H	CTTACGGCTCTCGCAGAAGA	GAACCGTGATCCAGGTTTTG	1100/930	55°C	-	A:1100 B:930	2	0.28	AY641730	flavanone 3-hydroxylase

*Note: “C” represents chloroplast CAPS markers, “M” represents mitochondria CAPS markers, “G” represents nuclear CAPS markers, and “PAL”, “F3H” represents nuclear STS markers.

^§^*PIC* Polymorphism Information Content.

^※^The primer pairs were referenced from (a) Kaundun and Matsumoto (2004) and (b) Kaundun and Matsumoto (2003).

A total of 98 alleles out of 39 polymorphic loci were detected in 55 germplasm. In 10 cytoplasmic CAPS loci, the average number of alleles was 2 and polymorphism information content (PIC) ranged from 0.13 (C02) to 0.35 (M06), with an average of 0.25 per locus. In 29 nuclear STS and CAPS markers, the number of alleles varied from 2 to 7, with an average of 2.7 per locus. The PIC values widely varied from 0.04 (G16) to 0.62 (G22), with an average of 0.34 per locus (Table 2).

### Identification of the prevailing tea cultivars in Taiwan

**Figure 1:**
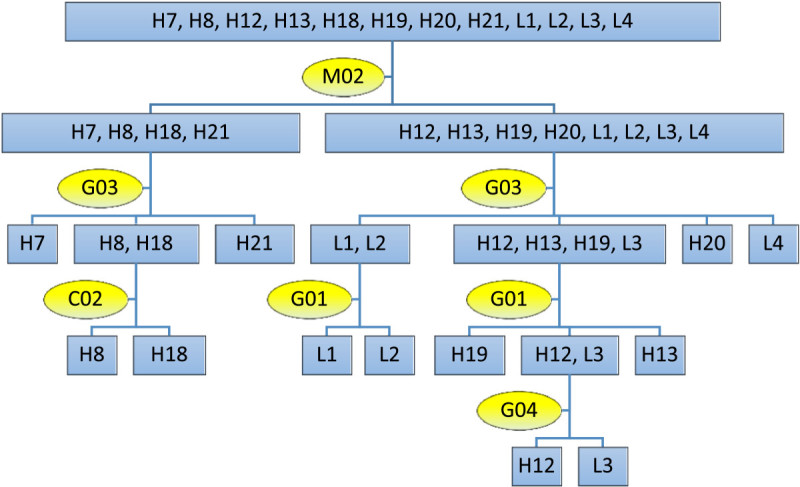
**The flow chart for identifying 12 prevailing tea cultivars in Taiwan.** The yellow circle frames represent marker codes, and the blue square frames represent cultivar codes. Cultivar and marker codes are shown in Tables 1 and 2. By using five core markers, 12 prevailing cultivars could be identified. M02 can be used to discriminate cultivars attributed to *sinensis* or *assamica* group. G03 and C02 are employed to identify four cultivars within the *assamica* group. Cultivars of the *sinensis* group can be distinguished by G03, G01 and G04.

Two STS and 37 CAPS markers developed in this study can be used to distinguish all 55 core germplasm in Taiwan, and their band patterns are shown in Additional file 1: Table S1. For the identification of 12 prevailing tea cultivars in Taiwan, the electrophoresis patterns of cleaved fragments in each STS and CAPS marker are shown in Additional file 1: Figure S2. In order to establish a flow chart for identifying 12 prevailing tea cultivars in Taiwan, five core markers, including M02 (mitochondria), C02 (chloroplast), G01 G03, and G04 (nuclear), were selected by variety-specific marker and PIC value. First, the *sinensis* type and the *assamica* type groups were distinguished by using the M02 marker. Secondly, the G03 and C02 can be employed to discriminate four cultivars within *assamica* type group, and the G03, G01 and G04 were used to separate eight cultivars within *sinensis* type group (Figure 1). In addition to five core markers, the remaining 34 markers could be used as a supplementary tool if more new varieties need to be identified in the future.

### Genetic diversity of tea germplasm in Taiwan

On the basis of taxonomy, 55 tea germplasm in Taiwan can be divided into three classifications, including *sinensis* type (S and SA), *assamica* type (A and AS) and wild species in Taiwan (F and FY) (Table 1). The average genetic distance among the three groups are shown in Table 3. The average genetic distance between *sinensis* type (S and SA) and wild species (F and FY) is 0.45, and that between *assamica* type (A and AS) and wild species (F and FY) is 0.47. Both distances are larger than that between *sinensis* type (S and SA) and *assamica* type (A and AS) (0.28). According to the genetic distance matrix of MRD coefficients among all 55 core germplasm (Table 4 and Additional file 1: Table S2), the average distance of wild species (0.25) is less than that of *sinensis* type (S and SA) (0.44) and *assamica* type (A and AS) (0.41). Based on two indices for estimating genetic variation within the populations, involving the observed number of alleles (N_A_) and effective number of alleles (N_e_), it showed that the *assamica* type (A and AS) (N_A_ = 2.34, N_e_ = 1.66) were more similar to *sinensis* type (S and SA) (N_A_ = 2.34, N_e_ = 1.77) compared to the wild species (F and FY) (N_A_ = 1.72, N_e_ = 1.24) (Table 4). It reveals that the genetic diversity within the cultivated species (*C. sinensis*) is higher than that within wild species in Taiwan (*C. formosensis*). Besides, larger parameters on the observed heterozygosity (H_O_), Nei’s gene diversity (H) and Shannon’s Information index (I), were detected in both *assamica* type (A and AS) (H_O_ = 0.35, H = 0.34, I = 0.55) and *sinensis* type (S and SA) (H_O_ = 0.38, H = 0.36, I = 0.58) compared to the wild species (F and FY) (H_O_ = 0.16, H = 0.15, I = 0.26) (Table 4). It also demonstrated that the cultivated species (*C. sinensis*) had greater genetic diversity than the wild species (*C. formosensis*).

**Table 3:** **The averages of genetic distance among the** ***sinensis*** **type (S and SA), the** ***assamica*** **type (A and AS) groups and the wild species in Taiwan (F and FY)**

Between groups	***Sinensis*** type and ***assamica*** type	***Sinensis*** type and wild species	***Assamica*** type and wild species
Genetic distance	0.28	0.45	0.47

**Table 4:** **The genetic diversity and genetic distance of different tea groups based on 10 cytoplasmic markers and 29 nuclear markers**

Group	N	Cytoplasmic markers			Nuclear markers					MRD		
		N_A_	N_e_	I	N_A_	N_e_	H_O_	H	I	Mean	Min	Max
A and AS	14	1.80	1.32	0.32	2.34	1.66	0.35	0.34	0.55	0.41	0.08	0.58
S and SA	33	1.70	1.48	0.39	2.34	1.77	0.38	0.36	0.58	0.44	0.11	0.62
F and FY	8	1.10	1.03	0.04	1.72	1.24	0.16	0.15	0.26	0.25	0.11	0.39
*C. sinensis*	47	2.00	1.49	0.49	2.62	1.79	0.38	0.37	0.62	0.47	0.08	0.65
Total	55	2.00	1.48	0.48	2.69	1.81	0.35	0.39	0.66	0.49	0.08	0.69

Note: N, No. of germplasm; N_A_, average observed number of alleles; N_e_, average effective number of alleles; H_O_, observed heterozygosity; H, Nei’s gene diversity; I, Shannon’s Information index; MRD, Modified Roger’s Distance; Mean, average MRD; Min, minimum MRD; Max, Maximum MRD.

### Cluster analysis and principle coordinates analysis of tea germplasm in Taiwan

The genetic distances between all pairwise combinations were listed in Additional file 1: Table S2. The values among 55 surveyed germplasm in Taiwan ranged from 0.08 to 0.69, with an average value of 0.49. Among the germplasm of 47 cultivated tea (*C. sinensis*), the average values was 0.47. If only 37 *sinensis* type tea (S and SA) were surveyed, the genetic distances among this group ranged from 0.11 (TTES-14 and TTES-15) to 0.62 (Chin-Shin-Oolong and TTES-17), with an average value of 0.44. As for 14 *assamica* type tea (A and AS), the genetic distances ranged from 0.08 (TTES-8 and Jaipuri; Shan-1 and Shan-2) to 0.58 (Shan and Manipuri), with an average value of 0.41. However, the genetic distances among eight wild species (F and FY) were relatively small, ranging from 0.11 (Long-Tou wild tea and Le-Ye wild tea; Ming-Hai wild tea and Nan-Fong wild tea) to 0.38 (De-Hua-She wild tea and Yung-Kang wild tea), with an average value of 0.25.

**Figure 2:**
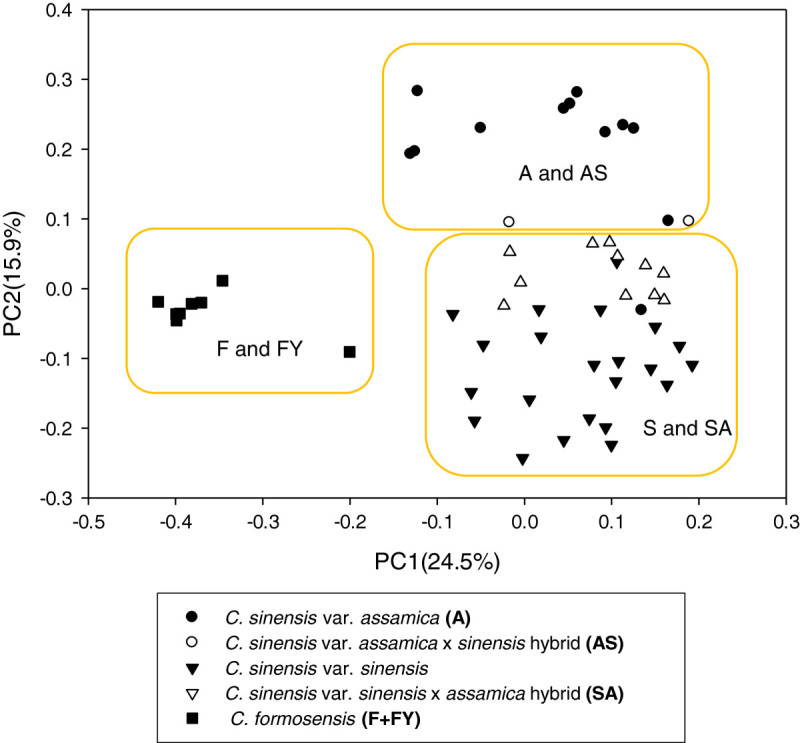
**Principal coordinate plots of 55 tea germplasm in Taiwan using 39 STS and CAPS loci based on modified Roger’s distance coefficient.** The cultivar codes are the same as Table 1. A and AS: the *assamica* type; S and SA: the *sinensis* type ; F and FY: the Taiwanese wild species. The components of the first dimension explaining 24.5% genetic diversity separated *C. formosensis* from the rest groups, and the components of the second dimension explaining 15.9% genetic diversity isolated *C. sinensis* var. *assamica* and *C. sinensis* var. *assamica* x var. *sinensis* hybrid from the other groups.

In PCoA based on MRD estimates of all 55 germplasm, the first, second and third principle coordinates (abbreviated to PC1, PC2 and PC3) explained 24.5%, 15.9% and 11.3% of the molecular variance, respectively, while the cumulative contribution was 51.8%. The first two principle coordinates were used to develop the PCoA plot shown in Figure 2. In the PC1, the 55 tea germplasm were divided into two major groups, cultivated tea (*C. sinensis*) and wild species in Taiwan (*C. formosensis*). In the PC2, the cultivated tea (*C. sinensis*) were divided into two major groups, *sinensis* type (S and SA) and *assamica* type (A and AS).

**Figure 3:**
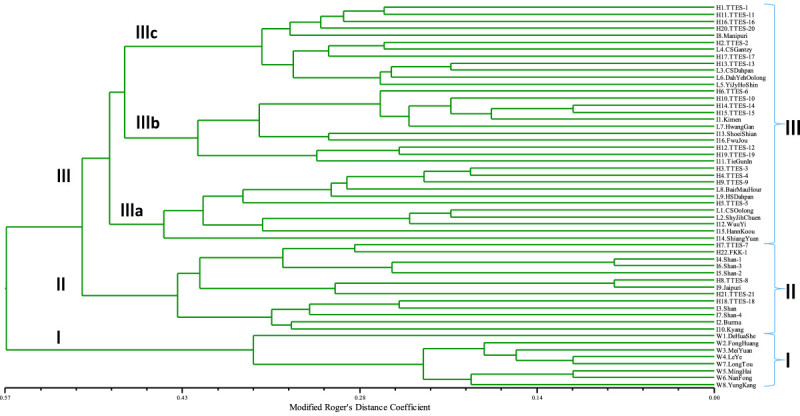
**Dendrogram of 55 tea germplasm in Taiwan using 39 STS and CAPS loci by UPGMA method based on modified Roger’s distance coefficient.** Three major groups were divided in this dendrogram. GroupIincluded *C. formosensis* (F) and *C. formosensis* var. *yungkangensis* (FY), groupII included *C. sinensis* var. *assamica* (A) and *C. sinensis* var. *assamica* × var. *sinensis* hybrid (AS), and groupIII included *C. sinensis* var. *sinensis* (S) and *C. sinensis* var. *sinensis* × var. *assamica* hybrid (SA). Three subgroups of groupIIIcomprised different introduced germplasm and their derived varieties.

The UPGMA dendrogram was constructed to separate the 55 germplasm into three major groups (Figure 3). Based on genetic distance coefficient of 0.57, the first group (GroupI) including *C. formosensis* could be isolated from the cultivated germplasm (*C. sinensis*). When the coefficient was reduced to 0.51, the *assamica* type (A and AS) (GroupII) and the *sinensis* type (S and SA) (GroupIII) germplasm were distinguished. Group III was divided into three subgroups, namely Group IIIa, Group IIIb, and Group IIIc. Many famous varieties belonged to Group IIIa including Chin-Shin-Oolong, Shy-Jih-Chuen, Bair-Mau-Hour, Wuu-Yi, Horng-Shin-Dahpan, and its derived varieties (TTES-3, TTES-4, and TTES-9). The Group IIIb comprised of Tiee-Guan-In, Hwang-Gan, and its derived varieties (TTES-10, TTES-12, TTES-14, TTES-15, and TTES-19). The Group IIIc, on the other hand, contained Chin-Shin-Gantzy, Chin-Shin-Dahpan and its derived variety (TTES-1), Dah-Yeh-Oolong, and its derived varieties (TTES-2, TTES-11, TTES-16, TTES-17, and TTES-20), Ying-Jy-Horng-Shin its derived variety (TTES-13).

## Discussion

### Polymorphism of STS and CAPS markers

In this study, 11 nuclear CAPS markers including G03, G12, G14, G15, G17, G18, G20, G22, G23 and G26 showed multi-allele patterns, while the others had only two alleles (bi-allele) (Table 2). There was only one restriction site within each CAPS locus resulting in the bi-allele markers, and their genotypes were easily scored and interpreted. Otherwise, the multi-allele markers were based on different point mutation positions within the locus that had more than two restriction sites. They yielded more complicated genotypes but may still be considered very useful. For example, the multi-allele CAPS markers could be used widely in pepper breeding for viral resistance (Yeam et al. 2005).

Polymorphism information content (PIC) means different informative levels of a locus and it also implies the genetic variation of a marker. The value larger than 0.5, ranging from 0.25 to 0.5, and smaller than 0.25 suggest that the locus is highly informative, reasonably informative, and slightly informative, respectively (Botstein et al. 1980). Of all the 39 cytoplasmic and nuclear markers examined in this study, the PIC ranged from 0.04 to 0.62, with an average of 0.32. The PIC of 10 cytoplasmic markers was 0.25, and seven of them were reasonably informative. Otherwise, the remaining three were slightly informative (Table 2). The 29 nuclear markers had an averaged PIC of 0.34, in which six were found to be highly informative, 16 were reasonably informative, and the remaining seven were slightly informative. The averaged PIC of the nuclear markers was higher than the cytoplasmic, and the average of the mtDNA markers (0.29) was higher than the cpDNA (0.18) (Table 2). Similar results were also reported by Ishii’s group, in which they found that the nuclear microsatellites (the averaged PIC is 0.89) had higher PIC values than the chloroplast microsatellites (the averaged PIC is 0.38) among A-genome species of rice (Ishii et al. 2001). Because the variation of cytoplasmic markers are lower than nuclear markers, the former could be used to examine relationship among distant-related taxa, and the latter are more suitable for the assessment of genetic diversity of close- related taxa.

In our previous study, the observed number of EST-SSR alleles (N_A_) per locus was 5.6 (Hu et al. 2011). However, in this study, the values of STS and CAPS markers derived from cytoplasmic and nuclear were 2.00 and 2.69, respectively. The PIC per locus for EST-SSR (0.62) was higher than those of STS and CAPS from cytoplasmic (0.25) and nuclear (0.34). Because small size difference between polymorphic bands was shown in the EST-SSR markers, there was high resolution of agarose gel, polyacrylamide gel electrophoresis or Genetic Analyzer (Hu et al. 2011). However, large size difference between polymorphic bands was found in STS or CAPS markers, and it suggested that only less expensive agarose gel was needed to obtain accurate data.

### Identification of 12 prevailing tea cultivars in Taiwan

In this study, 12 dominant cultivars were selected for variety identification based on the following criteria: (1) the acreage under cultivation of each variety; (2) the variety suitable for manufacturing unique tea; and (3) the newly bred varieties. According to statistics data from Tea Research and Extension Station in 2011, these 12 cultivars take over 98% acreage of Taiwan. Of these 12 cultivars, Chin-Shin-Oolong, TTES-12, Shy-Jih-Chuen, Chih-Shih-Dahpan, and TTES-13 are the top five cultivars in Taiwan that has been found to be suitable for both Paochong tea and Oolong tea. Shy-Jih-Chuen and Chih-Shih-Dahpan are mainly grown in Nantou County and north-west region of Taiwan, respectively, while others are distributed around Taiwan (estimated by Tea Research and Extension Station in 2011). Besides, Chin-Shin-Gantzy is fitted for green tea, and TTES-18, TTES-8, and TTES-7 are the excellent cultivars for making black tea. Chin-Shin-Gantzy is cultivated in New Taipei City, and the other three cultivars are mainly planted in Nantou County (estimated by Tea Research and Extension Station in 2011). In addition, varieties TTES-19 and TTES-20 were bred for manufacturing Paochong and Oolong tea, having been protected by the “Plant Variety and Plant Seed Act” in Taiwan since 2004 (Tsai et al. 2004a). TTES-21, on the other hand, was designated in 2008 for black tea procession (Chiu et al. 2009). These cultivars are most urgently desirable for variety identification in Taiwan.

Tea commercial products are manufactured through the application ofhigh temperature and the use of fermentation treatments at a panning step. These processes could eventually lead to dramatic DNA degradation. Additionally, tea merchants or farmers often blend the tea with different varieties to increase its flavor or reduce material cost. To solve the above problems, we have reported that DNA markers less than 1 kb are less affected by procession treatments and are useful for variety identification. Moreover, the chloroplast DNA markers with haploid genotypes and maternal inheritance could be effectively applied to identify the mixed-varieties of tea products (Hu et al. 2006). Since most STS and CAPS markers in this study are less than 850 bp, they may have application potential in identifying different varieties or mixed-varieties of processed tea.

### Genetic diversity of tea germplasm in Taiwan

The consistent results of germplasm classification were found in the principal coordinate analysis and cluster analysis. A total of 55 germplasm can be divided into three groups: *sinensis* type (S and SA), *assamica* type (A and AS) and Taiwan wild species (F and FY). The *sinensis* type (S and SA) and *assamica* type (A and AS) are generally called cultivated tea (*C. sinensis*). The former is a shrub with small leaves and can withstand cold climates; while the latter has tall tree-like structure with large leaves and is suitable for warm tropical climates (Banerjee 1992). Besides, the latter has more flavanols content so it was found to be more suitable for making black tea. Meanwhile, the *sinensis* type has been found to be suitable for manufacturing green tea or Oolong tea (Takeo 1992). In Taiwan, cultivated tea is mainly distributed in Nantou County (48.4%), Chiayi County (15.5%) and New Taipei City (10.6%) (Council of Agriculture 2012). The *assamica* type tea retains about 3.9% acreage which is mainly distributed in Nantou County, while the *sinensis* type is about 96.1% which is widely distributed in Taiwan (estimated by Tea Research and Extension Station in 2011). On the other hand, wild tea species is distributed in the central, southern and eastern regions of Taiwan. Various names have been given to the wild species, and *Camellia formosensis* is the official name based on the RPB2 (large sub-unit of RNA polymerase) gene of nuclear DNA sequence and morphological analyses (Su et al. 2007; Su et al. 2009). It can be well distinguished from cultivated tea (*C. sinensis*) by the glabrous ovaries and winter buds (Su et al. 2007). In this study, the results of both principal coordinate analysis and cluster analysis have supported that the wild species (*C. formosensis*) is monophyletic and independent from the cultivated tea (*C. sinensis*).

The genetic diversity can be accessed by many parameters. The N_A_ (observed number of alleles) is a count of the mean number of alleles with nonzero frequency across loci; the N_e_ (effective number of alleles) is an estimate of the mean number of equally frequent alleles in an ideal population; the H_o_ (observed heterozygosity) is an estimate proportion of observed heterozygotes at a given locus; the H (Nei’s gene diversity) is estimated proportion of expected heterozygotes under random mating; the I (Shannon information index) is an index as a measure of gene diversity (Yeh and Boyle 1997). According to the genetic diversity analysis, all parameters or indices showed that higher genetic diversity or genetic variation were detected in the *sinensis* type (S and SA) and the *assamica* type (A and AS) than wild species (Table 4). One possible explanation is that the cultivated tea (A, AS, S and SA) originated from diverse regions (China, Myanmar, Thailand, India, and so on) and had frequent inter-crossings. However, genetic recombination only occurred in a limited local wild species in Taiwan. This rationalization differs from that of Lai et al. (2001), in which they used RAPD and ISSR markers to evaluate the gene diversity of 37 tea samples in Taiwan. They reported that the native Taiwan wild species had the highest genetic diversity, followed by the *sinensis* type and the *assamica* type (Lai et al. 2001). There are two contrarieties that could be raised against this: first, two (Laitou and Shueijing wild tea) of six native Taiwan wild tea samples in Lai et al. (2001) are *C. furfuracea* instead of *C. formosensis* authenticated by Su (2007). This would lead to overestimate the diversity of native wild species. Second, all of three *assamica* varieties surveyed in Lai et al. (2001) merely originated from India, which are not representative of the tea wild species.

The tea industry in Taiwan began in the Jiaqing era of Ching Dynasty (AD 1796 to 1820), and a few tea varieties were introduced from China (Jun 1997). During the Japanese occupation period (AD 1896 to 1945), four landraces including Chin-Shin-Oolong, Dah-Yeh-Oolong, Chin-Shin-Dahpan, and Ying-Jy-Horng-Shin were recommended to the tea farmers. In addition, Hwang-Gan and Horng-Shin-Dahpan were also the prevailing cultivars at that time. Since 1945, the above six varieties have been used as female parents for hybridization breeding (Sanui 2011; Shyu and Juan 1993). According to cluster analysis in this study (Figure 3), Chin-Shin-Oolong and Horng-Shin-Dahpan belonged to Group IIIa, Hwang-Gan was classified in Group IIIb, and the remaining three landraces (Dah-Yeh-Oolong, Chin-Shin-Dahpan, Ying-Jy-Horng-Shin) were categorized in Group IIIc. However, all of these six varieties were introduced from Fukien or Guangdong of China (Sanui 2011).

Genetic vulnerability is a common problem in most of the tea-production countries, because only a few specific varieties are grown in large-scale and not many varieties have been used as the breeding parents (Yao et al. 2008). For example, a famous cultivar Yabukita contributes more than 80% of its tea acreage in Japan for making green tea (Kaundun and Matsumoto 2004). Besides, the other prevailing varieties including Kanayamidori, Sayamakaori, Saemidori, Okumidori, Meiryoku etc. were selected from Yabukita (Tanaka 2012). This could possibly lead some alleles to be eliminated and result in genetic erosion when most cultivars are replaced by a few varieties. Once the dramatically biotic or abiotic stress occurs, it is more likely to cause reduction in the production of the same or close-related cultivars, which could induce a crisis in the tea industry, leading to its possible collapse. In fact, a similar problem also exists in Taiwan. The top three prevailing cultivars in Taiwan take over 84.2% acreage including Chin-Shin-Oolong (57.3%), TTES-12 (13.7%) and Shy-Jih-Chuen (13.2%) (estimated by Tea Research and Extension Station in 2011). According to leaf morphological characters and ISSR DNA markers, a high similarity between Chin-Shin-Oolong and Shy-Jih-Chuen was found previously (Hu 2004). In this study, the genetic distance between these two cultivars (0.21) is far below the average (0.49) (Table 4 and Additional file 1: Table S2), and the alleles of all 10 cytoplasmic markers are identical (Additional file 1: Table S1). It was confirmed that Shy-Jih-Chuen originated from Chin-Shin-Oolong. In addition, these two cultivars accounting for 70.5% of all tea plantations in Taiwan, and Chin-Shin-Oolong is also the male parent of another two new varieties, TTES-19 and TTES-20, which were released in 2004 (Tsai et al. 2004a). In order to avoid the genetic vulnerability and increase the genetic diversity of tea varieties in Taiwan, the new parental lines could be referred to as the dendrogram of cluster analysis in this study (Figure 3). The elite parents from different geographical origins or genetic background could also be chosen.

## Conclusions

Tea is an important economic crop in Taiwan. Attributed to different eras and production areas, many unique types of tea have been expanded in the island, and accordingly, various genetic resources including introduced varieties, landraces, bred varieties and wild species were adopted. In order to develop a stable, fast and reliable marker system for variety identification and assessing genetic diversity of germplasm in Taiwan, 37 CAPS and two STS markers were successfully designed. Above all, five core markers have been found to be sufficient in identifying the prevailing varieties.

According to the genetic diversity analysis, principal coordinate analysis and cluster analysis on tea germplasm in Taiwan, three points of perception have been proposed. First, the high genetic diversity was found between the cultivated (*C. sinensis*) and wild species (*C. formosensis*) in Taiwan, although the genetic resources of wild species have not been used very well. Next, the genetic diversity of wild species among different areas of Taiwan was relatively small. Finally, the genetic relationship among the top prevailing cultivars is too close. Therefore, broadening the genetic diversity of the tea varieties is necessary for tea breeding in Taiwan.

## Electronic supplementary material

Additional file 1: Table S1:The band patterns of each STS and CAPS marker for all 55 core tea germplasm in Taiwan. **Table S2.** Matrix of genetic distance among pairs of 55 tea germplasm in Taiwan based on modified Roger’s distance coefficients. **Figure S1.** A. Partial nucleotide sequences of three cultivars amplified with G01 primer set, and arrow points indicated SNP sites. B. G01 CAPS marker designed from SNP sites of G01 primer set. **Figure S2.** The cleaved fragment patterns of each STS and CAPS marker for 12 prevailing tea cultivars in Taiwan. (DOCX 3 MB)

Below are the links to the authors’ original submitted files for images.

Authors’ original file for figure 1

Authors’ original file for figure 2

Authors’ original file for figure 3

## Abbreviations

AFLP: Amplified fragment length polymorphism
CAPS: Cleaved amplified polymorphic sequence
cpDNA: Chloroplast genome
EST: Expressed sequence tag
EST-SSR: Expressed sequence tag - simple sequence repeat
InDel: Insertion/deletion
ISSR: Inter-simple sequence repeat
MRD: Modified Roger’s distance
mtDNA: Mitochondria genome
PCoA: Principal coordinate analysis
PCR-RFLP: Polymerase chain reaction **-** restriction fragment length polymorphism
PIC: Polymorphism information content
RAPD: Randomly amplified polymorphic DNA
SNP: Single nucleotide polymorphism
STS: Sequence tagged site
UPGMA: Unweighted pair group method with arithmetic mean algorithm.

## References

[CR1] Bandyopadhyay T (2011). Molecular marker technology in genetic improvement of tea. Int J Plant Breed Genet.

[CR2] Banerjee B, Willson KC, Clifford MN (1992). Botanical classification of tea. Tea: cultivation to consumption.

[CR3] Barua PK (1963). Classification of the tea plant. Two and a Bud.

[CR4] Botstein D, White RL, Skolnick M, Davis RW (1980). Construction of a genetic linkage map in man using restriction fragment length polymorphisms. Am J Hum Genet.

[CR5] Chiu TF, Chiu TF, Wang CH (1988). Tea production and research in Taiwan. Recent development in tea production.

[CR6] Chiu CF, Lin JC, Huang CC, Lin JH, Shiau JH (2009). New cultivar of black tea - TTES No. 21. Taiwan Tea Res Bull.

[CR7] Collard BCY, Jahufer MZZ, Brouwer JB, Pang ECK (2005). An introduction to markers, quantitative trait loci (QTL) mapping and marker-assisted selection for crop improvement: the basic concepts. Euphytica.

[CR8] Council of Agriculture (2012). Agricultural statistics yearbook in 2011.

[CR9] Doyle JJ, Doyle JL (1990). Isolation of plant DNA from fresh tissue. Focus.

[CR10] Drenkard E, Glazebrook J, Preuss D, Ausubel FM, Caetano-Anolles G, Gresshoff PM (1997). Use of cleaved amplified polymorphic sequences (CAPS) for genetic mapping and typing. DNA markers: protocols, applications, and overviews.

[CR11] Gunasekare MTK (2007). Applications of molecular markers to the genetic improvement of *Camellia sinensis* L. (tea) - a review. J Hortic Sci Biotechnol.

[CR12] Hu CY (2004). Studies on the variations in leaf characters and DNA sequences of tea germplasm in Taiwan.

[CR13] Hu CY, Tsai YZ, Lin SF (2005). Using ISSR DNA markers to evaluate genetic diversity of tea germplasm in Taiwan. J Agri Assoc China.

[CR14] Hu CY, Tsai YZ, Lin SF (2006). Evaluating the feasibility of molecular identification for made tea varieties. J Agri Assoc China.

[CR15] Hu CY, Lin YC, Hsieh WT, Tseng YH, Lin SF, Tsai YZ (2011). Using EST-SSR markers to identify tea (*Camellia sinensis*) cultivars in Taiwan. Taiwan Tea Res Bull.

[CR16] Ishii T, McCouch SR, Xu Y (2001). Nuclear- and chloroplast-microsatellite variation in A-genome species of rice. Genome.

[CR17] Jones N, Ougham H, Thomas H, Pasakinskiene I (2009). Markers and mapping revisited: finding your gene. New Phytol.

[CR18] Jun IM (1997). The culture and science of tea in Taiwan. Taiwan Tea Res Bull.

[CR19] Jun IM, Lin ML (1997). Present status of tea industry in Taiwan. Taiwan Tea Res Bull.

[CR20] Katoh Y, Katoh M, Takeda Y, Omori M (2003). Genetic diversity within cultivated teas based on nucleotide sequence comparison of ribosomal RNA maturase in chloroplast DNA. Euphytica.

[CR21] Kaundun SS, Matsumoto S (2002). Heterologous nuclear and chloroplast microsatellite amplification and variation in tea, *Camellia sinensis*. Genome.

[CR22] Kaundun SS, Matsumoto S (2003). Identification of processed Japanese green tea based on polymorphisms generated by STS-RFLP analysis. J Agric Food Chem.

[CR23] Kaundun SS, Matsumoto S (2004). PCR-based amplicon length polymorphisms (ALPs) at microsatellite loci and indels from non-coding DNA regions of cloned genes as a means of authenticating commercial Japanese green teas. J Sci Food Agric.

[CR24] Kaundun S, Matsumoto S (2011). Molecular evidence for maternal inheritance of the chloroplast genome in tea, *Camellia sinensis* (L.) O. Kuntze. J Sci Food Agric.

[CR25] Konieczny A, Ausubel FM (1993). A procedure for mapping Arabidopsis mutations using co-dominant ecotype-specific PCR-based markers. Plant J.

[CR26] Lai JA, Yang WC, Hsiao JY (2001). An assessment of genetic relationships in cultivated tea clones and native wild tea in Taiwan using RAPD and ISSR maskers. Bot Bull Acad Sin.

[CR27] Lin SY, Chen IZ, Tsai CM, Chen YL (2005). Detection of genetic relationship in Taiwan tea variety (*Camellia sinensis* (L.) O. Kuntze) with RAPD markers. J Chinese Soc Hort Sci.

[CR28] Liu K, Muse SV (2005). PowerMarker: an integrated analysis environment for genetic marker analysis. Bioinformatics.

[CR29] Miller MP (1997). TFPGA (Tools for population genetic analyses) ver. 1.3.

[CR30] Olson M, Hood L, Cantor C, Botstein D (1989). A common language for physical mapping of the human genome. Science.

[CR31] Palmer JD, Soltis PS, Soltis DE, Doyle JJ (1992). Mitichondrial DNA in plant Systematics: applications and limitations. Molecular systematics of plants.

[CR32] Powell W, Machray GC, Provan J (1996). Polymorphism revealed by simple sequence repeats. Trends Plant Sci.

[CR33] Rohlf FJ (1997). NTSYS-pc: numerical taxonomy and multivariate analysis system.

[CR34] Rozen S, Skaletsky HJ, Krawetz S, Misener S (2000). Primer3 on the WWW for general users and for biologist programmers. Bioinformatics methods and protocols: methods in molecular biology.

[CR35] Sanui H, Shyu YS (2011). Tea breeding in Taiwan. Tea breeding in Taiwan during Japanese occupation period.

[CR36] Schaal BA, Olsen KM (2000). Gene genealogies and population variation in plants. Proc Natl Acad Sci USA.

[CR37] Shu Y, Li Y, Zhu Y, Zhu Z, Lv D, Bai X, Cai H, Ji W, Guo D (2010). Genome-wide identification of intron fragment insertion mutations and their potential use as SCAR molecular markers in the soybean. Theor Appl Genet.

[CR38] Shyu YS, Juan IM (1993). Retrospect of tea breeding in Taiwan. Taiwan Tea Res Bull.

[CR39] Su MH (2007). Taxonomic study of Camellia formosensis (Masamune et Suzuki) M. H. Su, C. F. Hsieh et C. H. Tsou (Theaceae).

[CR40] Su MH, Tsou CH, Hsieh CF (2007). Morphological comparisons of Taiwan native wild tea plant (*Camellia sinensis* (L.) O. Kuntze forma formosensis Kitamura) and two closely related taxa using numerical methods. Taiwania.

[CR41] Su MH, Hsieh CF, Tsou CH (2009). The confirmation of Camellia formosensis (Theaceae) as an independent species based on DNA equence analyses. Bot Stud.

[CR42] Takeo T, Willson KC, Clifford MN (1992). Green and semi-fermented teas. Tea: cultivation to consumption.

[CR43] Tanaka J, Chen L, Apostolides Z, Chen Z-M (2012). Japanese tea breeding history and the future perspective. Global tea breeding: achievements, challenges and perspectives.

[CR44] Thiel T, Kota R, Grosse I, Stein N, Graner A (2004). SNP2CAPS: a SNP and INDEL analysis tool for CAPS marker development. Nucleic Acids Res.

[CR45] Tsai HT, Tsai IC, Liaw WR, Chang CK, Wang YW (2003). Study on the genetic diversity among the selected Taiwan tea cultivars/ lines using AFLP and RAPD markers. Taiwan Tea Res Bull.

[CR46] Tsai CM, Chang CK, Chen IZ, Chen KR, Tsai YZ, Chiou CF, Lin IC, Fan HJ (2004). Breeding report of two 2004 registered new tea cultivar TTES No. 19 and 20. Taiwan Tea Res Bull.

[CR47] Tsai YS, Liu SL, Wang HF, Ou SM (2004). Comparison of catechins contents and antioxidant activity of green teas from Taiwan major tea cultural cultivars. Taiwan Tea Res Bull.

[CR48] Wright S (1978). Evolution and the genetics of populations., vol 4. Variability within and among natural populations.

[CR49] Yao MZ, Chen L, Liang YR (2008). Genetic diversity among tea cultivars from China, Japan and Kenya revealed by ISSR markers and its implication for parental selection in tea breeding programmes. Plant Breed.

[CR50] Yeam I, Faber N, Jahn MM, Frantz JD, Kang BC, Lindeman W (2005). Allele-specific CAPS markers based on point mutations in resistance alleles at the pvr1 locus encoding eIF4E in Capsicum. Theor Appl Genet.

[CR51] Yeh FC, Boyle TJB (1997). Population genetic analysis of co-dominant and dominant markers and quantitative traits. Belg J Bot.

