# Supplementary material for: Development of STS and CAPS markers for variety identification and genetic diversity analysis of tea germplasm in Taiwan
Source: Bot Stud. 2014 Feb 1;55:12. doi: 10.1186/1999-3110-55-12 (PMC5430312; doi:10.1186/1999-3110-55-12)
Supplement: Supplementary file 1 — Additional file 1: Table S1:The band patterns of each STS and CAPS marker for all 55 core tea germplasm in Taiwan. Table S2. Matrix of genetic distance among pairs of 55 tea germplasm in Taiwan based on modified Roger’s distance coefficients. Figure S1. A. Partial nucleotide sequences of three cultivars amplified with G01 primer set, and arrow points indicated SNP sites. B. G01 CAPS marker designed from SNP sites of G01 primer set. Figure S2. The cleaved fragment patterns of each STS and CAPS marker for 12 prevailing tea cultivars in Taiwan. (DOCX 3 MB) [file 40529_2013_67_MOESM1_ESM.docx]

**Additional file Table S1** The band patterns of each STS and CAPS marker for all 55 core tea germplasm in Taiwan

| Marker^#^ | Prevailing Tea Cultivar Code^§^ | | | | | | | | | | |
| --- | --- | --- | --- | --- | --- | --- | --- | --- | --- | --- | --- |
|  | H1 | H2 | H3 | H4 | H5 | H6 | H7 | H8 | H9 | H10 | H11 |
| C01 | A | A | A | A | A | A | A | A | A | A | A |
| C02 | B | B | B | B | B | B | B | B | B | B | B |
| C03 | B | B | A | A | A | B | B | B | A | B | B |
| M01 | A | A | A | A | A | A | A | A | A | A | A |
| M02 | B | B | B | B | B | B | A | A | B | B | B |
| M03 | A | A | B | B | B | B | B | B | B | B | A |
| M04 | A | A | B | B | B | B | B | B | B | B | A |
| M05 | A | A | A | A | A | B | A | A | A | B | A |
| M06 | B | B | B | B | B | A | B | B | B | A | B |
| M07 | B | B | A | A | A | B | B | B | A | B | B |
| G01 | AA | AA | AA | AA | AA | AA | AA | AA | AA | AA | AA |
| G02 | AB | AB | AB | AA | AB | AA | AB | AB | AB | AB | AB |
| G03 | AB | BC | AB | AB | AC | AB | CC | BB | AB | AB | AB |
| G04 | AB | AB | AA | AA | AB | AA | AB | AB | AA | AA | AB |
| G05 | BB | BB | BB | BB | BB | BB | BB | AB | BB | BB | BB |
| G06 | AA | AB | AA | AA | AB | AA | AB | AA | AA | AA | AA |
| G07 | BB | AA | BB | BB | AB | BB | AA | BB | BB | BB | BB |
| G08 | AA | AA | AA | AA | AA | AA | AA | AB | AA | AA | AA |
| G09 | BB | BB | BB | BB | BB | AB | BB | BB | BB | BB | BB |
| G10 | AA | AA | AA | AA | AA | AA | AA | AB | AA | AA | AA |
| G11 | BB | AB | BB | BB | BB | BB | BB | BB | BB | BB | BB |
| G12 | AB | AB | AB | AB | BB | BB | AB | AC | AB | AB | AB |
| G13 | BB | BB | BB | BB | BB | BB | BB | BB | BB | BB | BB |
| G14 | FF | FF | FF | FF | AF | AF | AF | AF | AF | FF | FF |
| G15 | AA | CD | AC | AC | BC | AC | DD | CC | AC | AC | AA |
| G16 | BB | BB | BB | BB | BB | BB | BB | BB | BB | BB | BB |
| G17 | BB | BB | BB | BB | AB | AB | BB | BC | AB | BB | BB |
| G18 | BB | BB | BC | BB | AA | AB | AB | BB | BB | BB | AB |
| G19 | AB | AB | AB | AA | AA | AB | AA | AB | AA | AA | AB |
| G20 | CC | CC | CC | CC | CC | CC | CC | AC | CC | CC | CC |
| G21 | AB | BB | AB | AB | AB | AB | AB | BC | BB | BB | BB |
| G22 | AA | AC | AC | AA | DD | BD | BC | AA | AC | AB | CD |
| G23 | CC | BC | BC | CC | AA | AC | AB | CC | BC | CC | AB |
| G24 | BB | AA | AB | AB | AB | AB | AB | AB | AB | AB | AB |
| G25 | BB | BB | BB | BB | BB | BB | BB | AB | BB | BB | BB |
| G26 | DD | CD | DD | CD | DD | BD | DD | AC | BC | CD | CD |
| G27 | AB | AB | AB | AB | AB | BB | AA | AB | AA | AA | AB |
| PAL | AA | AA | AA | AA | AA | AA | AA | AB | AA | AA | AA |
| F3H | BB | BB | BB | BB | AA | AB | AB | BB | BB | BB | AB |
| C01 | A | A | A | A | A | A | B | A | A | A | A |
| C02 | B | B | B | B | B | B | A | B | B | B | B |
| C03 | B | B | B | B | B | B | B | B | B | B | A |
| M01 | A | A | A | A | A | A | B | A | A | A | A |
| M02 | B | B | B | B | B | B | A | B | B | A | A |
| M03 | B | A | B | B | A | A | B | B | A | B | B |
| M04 | B | A | B | B | A | A | B | B | A | B | B |
| M05 | B | A | B | B | A | A | A | B | A | A | A |
| M06 | A | B | A | A | B | B | B | A | B | B | B |
| M07 | B | B | B | B | B | B | B | B | B | B | B |
| G01 | AB | AA | AA | AA | AA | AA | AA | BB | AB | AA | AA |
| G02 | AB | AB | AB | AB | AB | BB | AB | AB | AA | AB | BB |
| G03 | AC | AC | AB | AB | BB | BC | BB | AC | AB | BC | BC |
| G04 | BB | BB | AA | AB | AA | AB | AB | AB | AB | AB | AA |
| G05 | AB | AB | BB | BB | BB | BB | AB | BB | AB | AB | BB |
| G06 | AB | AB | AA | AA | AA | AB | AA | AB | AA | AB | AB |
| G07 | AB | AB | BB | BB | BB | AB | BB | AB | BB | AB | AB |
| G08 | AA | AA | AA | AA | AA | AA | AA | AA | AA | AA | AA |
| G09 | BB | BB | BB | BB | BB | BB | BB | BB | AB | BB | BB |
| G10 | AA | AA | AA | AA | AA | AA | AB | AA | AA | AA | AA |
| G11 | BB | AB | BB | BB | BB | AB | BB | BB | BB | BB | BB |
| G12 | BB | BB | AB | BB | BB | AB | AC | BB | BB | BC | AB |
| G13 | BB | BB | BB | BB | AB | AB | BB | BB | BB | BB | BB |
| G14 | FF | FF | FF | FF | FF | FF | AG | FF | FF | FF | FF |
| G15 | BC | BC | AC | AC | CD | CD | CC | BC | AC | CD | CD |
| G16 | BB | BB | BB | BB | BB | BB | BB | BB | BB | BB | BB |
| G17 | BB | BB | BB | BB | BB | BB | BC | BB | BB | BC | BB |
| G18 | BC | AC | AB | AB | AB | AB | BB | CC | AC | AB | AB |
| G19 | BB | AB | AA | AA | AB | AA | AB | BB | BB | AB | AA |
| G20 | CC | CC | CC | CC | CC | CC | AC | DD | CC | AC | CC |
| G21 | AA | AB | BB | BB | AB | AA | BC | AA | AA | BC | BB |
| G22 | AC | BC | AD | AD | BC | AD | AA | CC | BC | AD | CD |
| G23 | BC | AB | AC | AC | AB | AC | CC | BB | AB | AC | AB |
| G24 | AA | AA | AB | AB | BB | AB | AB | AA | AB | AB | AB |
| G25 | BB | BB | BB | BB | BB | BB | AB | BB | BB | AB | BB |
| G26 | DD | DD | BC | BC | CD | BC | BD | DD | DD | BD | DD |
| G27 | AB | AB | AB | AB | BB | AB | AB | AB | AB | BB | AB |
| PAL | AA | AA | AA | AA | AA | AA | AB | AA | AA | AB | AA |
| F3H | BB | AB | AB | AB | AB | AB | BB | BB | AB | AB | AB |
| C01 | A | A | A | A | A | A | A | A | A | A | B |
| C02 | B | B | B | B | B | B | B | B | B | B | A |
| C03 | A | A | B | B | B | B | B | A | A | B | B |
| M01 | A | A | A | A | A | A | A | A | A | A | B |
| M02 | B | B | B | B | B | B | B | B | B | B | A |
| M03 | B | B | A | A | A | A | B | B | B | B | B |
| M04 | B | B | A | A | A | A | B | B | B | B | B |
| M05 | A | A | A | A | A | A | B | A | A | B | A |
| M06 | B | B | B | B | B | B | A | B | B | A | B |
| M07 | A | A | B | B | B | B | B | A | A | B | A |
| G01 | BB | AB | AB | AB | AA | AA | AA | AA | AB | AA | AA |
| G02 | AA | AA | AB | BB | BB | AB | AB | AB | AB | AB | AB |
| G03 | AA | AA | AC | BC | AA | AA | AA | AB | AA | AB | BC |
| G04 | AA | AA | AB | AB | BB | AB | AA | AB | AA | AA | AA |
| G05 | BB | BB | BB | BB | BB | BB | BB | BB | BB | BB | BB |
| G06 | AA | AA | AB | AB | BB | AB | AB | AA | AB | AA | AA |
| G07 | BB | BB | AB | AB | AA | AB | AB | BB | AB | BB | AB |
| G08 | AA | AA | AA | AA | AA | AA | AA | AA | AA | AA | AA |
| G09 | BB | BB | BB | BB | BB | BB | BB | BB | BB | BB | BB |
| G10 | AA | AA | AA | AA | AA | AA | AA | AA | AA | AA | AB |
| G11 | BB | BB | AB | BB | AB | AB | BB | BB | BB | BB | BB |
| G12 | BB | BB | BB | BB | BB | BB | BB | BB | BB | BB | AA |
| G13 | BB | BB | BB | AB | BB | BB | BB | AB | BB | BB | BB |
| G14 | AF | AG | AF | AF | AF | FF | FF | FF | AF | FF | EF |
| G15 | AA | AA | BC | CD | BC | BC | BC | AC | BC | AC | CE |
| G16 | BB | AB | BB | BB | BB | BB | BB | AB | BB | BB | BB |
| G17 | AB | AB | AB | BB | BB | AB | BB | BB | AB | BB | BB |
| G18 | CC | AC | AC | BB | AC | AB | AB | AB | BC | AB | BB |
| G19 | BB | BB | BB | AB | AA | AB | AA | AB | AB | AA | AB |
| G20 | DD | DD | CC | CC | CC | CC | CC | CC | CC | CC | CC |
| G21 | AB | AB | BB | BB | AB | AB | BB | BB | AB | BB | AB |
| G22 | CC | BC | BC | AA | CD | AD | BD | AD | AC | BD | AC |
| G23 | BB | AB | AB | CC | AB | AC | AC | AC | BC | AC | BC |
| G24 | AA | AA | AA | AB | AA | AB | AA | AA | AA | AB | AA |
| G25 | BB | BB | BB | BB | BB | BB | AB | BB | BB | BB | BB |
| G26 | BD | BD | DD | DD | DD | DD | BD | BD | BB | DD | DD |
| G27 | AB | AB | AB | AB | BB | BB | AB | BB | AB | AB | AA |
| PAL | AA | AA | AA | AA | AA | AA | AA | AA | AA | AA | AA |
| F3H | BB | AB | AB | BB | AB | AB | AB | AB | BB | AB | BB |
| C01 | B | A | A | A | B | A | A | B | A | A | A |
| C02 | A | B | B | B | A | B | B | B | B | B | B |
| C03 | B | B | B | B | B | B | B | B | B | A | B |
| M01 | B | A | A | A | B | A | A | B | A | A | A |
| M02 | A | A | A | A | A | B | A | A | B | B | A |
| M03 | B | B | B | B | B | A | B | B | B | B | B |
| M04 | B | B | B | B | B | A | B | B | B | B | B |
| M05 | A | A | A | A | A | A | A | A | B | A | B |
| M06 | B | B | B | B | B | B | B | B | A | B | A |
| M07 | B | B | B | B | B | B | B | B | B | A | B |
| G01 | AA | AA | AA | AA | AA | BB | AA | AA | BB | AB | AB |
| G02 | AA | AA | AB | AA | AB | BB | AB | AB | BB | AA | AB |
| G03 | AA | BC | BB | CC | BC | BB | BB | AB | BC | AA | AB |
| G04 | AB | AA | AA | AA | AB | AA | AB | AA | AA | BB | AB |
| G05 | AB | BB | BB | BB | AB | BB | AB | BB | BB | BB | AB |
| G06 | AA | AA | AA | AA | AA | AA | AA | AA | AB | AA | AA |
| G07 | BB | AA | AB | AA | AB | BB | BB | BB | AB | BB | BB |
| G08 | AA | AA | AA | AA | AA | AA | AB | AA | AA | AA | AA |
| G09 | BB | BB | BB | BB | BB | BB | BB | BB | BB | BB | AB |
| G10 | AA | AA | AA | AA | AA | AA | AB | AA | AA | AA | AA |
| G11 | BB | BB | BB | BB | BB | BB | BB | BB | BB | BB | BB |
| G12 | AC | AA | AA | AA | AB | AB | AC | AA | BB | BB | AB |
| G13 | BB | BB | BB | BB | BB | BB | BB | BB | BB | BB | AB |
| G14 | AF | EG | EG | EG | BD | FF | AF | EF | AF | AF | AF |
| G15 | CC | DD | CC | DD | CD | CC | CC | AC | CC | AC | AC |
| G16 | BB | BB | BB | BB | BB | BB | BB | BB | BB | BB | BB |
| G17 | BC | BB | BB | BB | BB | BB | BC | BB | BB | AB | AB |
| G18 | BB | BB | BB | BB | BB | BB | BB | BB | CC | CC | AB |
| G19 | AA | AA | AA | AA | AA | BB | AB | AB | BB | BB | AB |
| G20 | AC | CC | CC | CC | CC | CC | AC | CC | CC | DD | DD |
| G21 | BC | BB | BB | BB | BB | AB | BC | BB | BB | AB | AB |
| G22 | AC | AC | AA | AC | AC | CC | AA | AA | CC | CC | AD |
| G23 | BC | BC | CC | BC | BC | BB | CC | CC | BB | BB | AC |
| G24 | AB | BB | BB | BB | BB | AB | AB | AB | AA | AA | AA |
| G25 | AB | BB | BB | BB | BB | BB | AB | BB | BB | BB | BB |
| G26 | BD | DD | DD | DD | DD | CD | BC | CD | BD | BB | CD |
| G27 | AB | AB | AB | AB | AB | AB | AB | AB | AB | AB | AB |
| PAL | AB | AA | AA | AA | AA | AA | AB | AA | AA | AA | AA |
| F3H | BB | BB | BB | BB | BB | BB | BB | BB | BB | BB | AB |
| C01 | A | A | A | A | A | A | A | A | A | A | A |
| C02 | B | B | B | B | B | B | B | B | B | B | B |
| C03 | A | B | B | B | B | B | B | B | B | B | B |
| M01 | A | A | A | A | A | A | A | A | A | A | A |
| M02 | B | B | B | B | B | B | B | B | B | B | B |
| M03 | B | B | B | B | B | B | B | B | B | B | B |
| M04 | B | B | B | B | B | B | B | B | B | B | B |
| M05 | A | A | B | B | B | B | B | B | B | B | A |
| M06 | B | B | A | A | A | A | A | A | A | A | A |
| M07 | A | A | B | B | B | B | B | B | B | B | B |
| G01 | AB | BB | AB | AA | AA | AA | AA | AA | AA | AA | AA |
| G02 | BB | AB | AA | AA | AA | AA | AA | AA | AA | AA | AA |
| G03 | CC | AA | AB | AB | BB | BB | BB | BB | BB | BB | BB |
| G04 | AB | AA | AB | AB | BB | BB | BB | BB | BB | BB | BB |
| G05 | BB | BB | AB | AB | AA | AA | AA | AA | AA | AA | AA |
| G06 | BB | AB | AA | AA | AA | AA | AA | AA | AA | AA | AA |
| G07 | AA | AB | BB | BB | BB | BB | BB | BB | BB | BB | BB |
| G08 | AA | AA | AA | AB | AB | AB | AB | AB | AB | AB | AB |
| G09 | BB | BB | AB | BB | BB | BB | BB | BB | BB | BB | BB |
| G10 | AA | AA | AA | AA | AB | BB | AB | AA | AA | AA | AB |
| G11 | BB | BB | BB | BB | BB | BB | BB | BB | BB | BB | BB |
| G12 | BB | BB | BB | BC | BB | BC | BB | BB | BC | BB | BC |
| G13 | AB | BB | BB | BB | BB | BB | BB | BB | BB | BB | BB |
| G14 | AF | AF | FF | AG | CC | AA | AA | AA | AA | AA | AA |
| G15 | DD | BC | AC | AA | CC | CC | CC | CC | CC | CC | CC |
| G16 | BB | BB | BB | BB | BB | BB | BB | BB | BB | BB | BB |
| G17 | BC | BC | AB | BC | BB | BB | BB | BB | BB | BB | BB |
| G18 | BC | AC | AC | BC | BB | BB | BB | BB | BB | BB | BB |
| G19 | AB | BB | BB | BB | BB | BB | BB | BB | BB | BB | BB |
| G20 | AD | AD | DD | AC | BB | BB | BB | BB | BB | BB | BB |
| G21 | AC | AC | AB | AC | CC | CC | CC | CC | CC | CC | CC |
| G22 | BC | BC | BC | AC | AA | AA | AA | AA | AA | AA | AA |
| G23 | BC | AB | AB | BC | CC | CC | CC | CC | CC | CC | CC |
| G24 | AA | AA | AB | AA | AA | AA | AA | AB | AA | AA | AA |
| G25 | AB | BB | BB | AB | AB | AB | AB | AB | AB | AB | AB |
| G26 | BD | DD | DD | BD | BB | BB | BB | DD | DD | BB | DD |
| G27 | AB | AB | AB | AB | BB | BB | AB | BB | BB | BB | BB |
| PAL | AB | AB | AA | AB | BB | BB | BB | BB | BB | BB | BB |
| F3H | BB | AB | AB | BB | BB | BB | BB | BB | BB | BB | BB |

Note §：Cultivar codes are the same as Table 1.

Note #：Marker codes are shown in Table 2.

**Additional file Table S2.** Matrix of genetic distance among pairs of 55 tea germplasm in Taiwan based on modified Roger’s distance coefficients

|  | **H1** | **H2** | **H3** | **H4** | **H5** | **H6** | **H7** | **H8** | **H9** | **H10** | **H11** | **H12** | **H13** | **H14** | **H15** | **H16** | **H17** | **H18** | **H19** | **H20** | **H21** | **H22** | **L1** | **L2** | **L3** | **L4** | **L5** | **L6** | **L7** | **L8** | **L9** | **I1** | **I2** | **I3** | **I4** | **I5** | **I6** | **I7** | **I8** | **I9** | **I10** | **I11** | **I12** | **I13** | **I14** | **I15** | **I16** | **W1** | **W2** | **W3** | **W4** | **W5** | **W6** | **W7** | **W8** |
| --- | --- | --- | --- | --- | --- | --- | --- | --- | --- | --- | --- | --- | --- | --- | --- | --- | --- | --- | --- | --- | --- | --- | --- | --- | --- | --- | --- | --- | --- | --- | --- | --- | --- | --- | --- | --- | --- | --- | --- | --- | --- | --- | --- | --- | --- | --- | --- | --- | --- | --- | --- | --- | --- | --- | --- |
| **H1** | 0.00 |  |  |  |  |  |  |  |  |  |  |  |  |  |  |  |  |  |  |  |  |  |  |  |  |  |  |  |  |  |  |  |  |  |  |  |  |  |  |  |  |  |  |  |  |  |  |  |  |  |  |  |  |  |  |
| **H2** | 0.34 | 0.00 |  |  |  |  |  |  |  |  |  |  |  |  |  |  |  |  |  |  |  |  |  |  |  |  |  |  |  |  |  |  |  |  |  |  |  |  |  |  |  |  |  |  |  |  |  |  |  |  |  |  |  |  |  |
| **H3** | 0.38 | 0.43 | 0.00 |  |  |  |  |  |  |  |  |  |  |  |  |  |  |  |  |  |  |  |  |  |  |  |  |  |  |  |  |  |  |  |  |  |  |  |  |  |  |  |  |  |  |  |  |  |  |  |  |  |  |  |  |
| **H4** | 0.38 | 0.45 | 0.20 | 0.00 |  |  |  |  |  |  |  |  |  |  |  |  |  |  |  |  |  |  |  |  |  |  |  |  |  |  |  |  |  |  |  |  |  |  |  |  |  |  |  |  |  |  |  |  |  |  |  |  |  |  |  |
| **H5** | 0.53 | 0.51 | 0.38 | 0.41 | 0.00 |  |  |  |  |  |  |  |  |  |  |  |  |  |  |  |  |  |  |  |  |  |  |  |  |  |  |  |  |  |  |  |  |  |  |  |  |  |  |  |  |  |  |  |  |  |  |  |  |  |  |
| **H6** | 0.45 | 0.50 | 0.42 | 0.43 | 0.44 | 0.00 |  |  |  |  |  |  |  |  |  |  |  |  |  |  |  |  |  |  |  |  |  |  |  |  |  |  |  |  |  |  |  |  |  |  |  |  |  |  |  |  |  |  |  |  |  |  |  |  |  |
| **H7** | 0.48 | 0.40 | 0.45 | 0.48 | 0.41 | 0.48 | 0.00 |  |  |  |  |  |  |  |  |  |  |  |  |  |  |  |  |  |  |  |  |  |  |  |  |  |  |  |  |  |  |  |  |  |  |  |  |  |  |  |  |  |  |  |  |  |  |  |  |
| **H8** | 0.45 | 0.47 | 0.45 | 0.42 | 0.56 | 0.49 | 0.47 | 0.00 |  |  |  |  |  |  |  |  |  |  |  |  |  |  |  |  |  |  |  |  |  |  |  |  |  |  |  |  |  |  |  |  |  |  |  |  |  |  |  |  |  |  |  |  |  |  |  |
| **H9** | 0.43 | 0.45 | 0.24 | 0.23 | 0.40 | 0.45 | 0.46 | 0.43 | 0.00 |  |  |  |  |  |  |  |  |  |  |  |  |  |  |  |  |  |  |  |  |  |  |  |  |  |  |  |  |  |  |  |  |  |  |  |  |  |  |  |  |  |  |  |  |  |  |
| **H10** | 0.39 | 0.44 | 0.38 | 0.36 | 0.52 | 0.32 | 0.46 | 0.43 | 0.37 | 0.00 |  |  |  |  |  |  |  |  |  |  |  |  |  |  |  |  |  |  |  |  |  |  |  |  |  |  |  |  |  |  |  |  |  |  |  |  |  |  |  |  |  |  |  |  |  |
| **H11** | 0.27 | 0.31 | 0.39 | 0.43 | 0.45 | 0.42 | 0.44 | 0.48 | 0.42 | 0.42 | 0.00 |  |  |  |  |  |  |  |  |  |  |  |  |  |  |  |  |  |  |  |  |  |  |  |  |  |  |  |  |  |  |  |  |  |  |  |  |  |  |  |  |  |  |  |  |
| **H12** | 0.47 | 0.45 | 0.44 | 0.49 | 0.51 | 0.39 | 0.46 | 0.51 | 0.53 | 0.40 | 0.49 | 0.00 |  |  |  |  |  |  |  |  |  |  |  |  |  |  |  |  |  |  |  |  |  |  |  |  |  |  |  |  |  |  |  |  |  |  |  |  |  |  |  |  |  |  |  |
| **H13** | 0.39 | 0.30 | 0.45 | 0.51 | 0.43 | 0.48 | 0.41 | 0.54 | 0.52 | 0.51 | 0.31 | 0.39 | 0.00 |  |  |  |  |  |  |  |  |  |  |  |  |  |  |  |  |  |  |  |  |  |  |  |  |  |  |  |  |  |  |  |  |  |  |  |  |  |  |  |  |  |  |
| **H14** | 0.42 | 0.45 | 0.40 | 0.38 | 0.45 | 0.25 | 0.46 | 0.45 | 0.38 | 0.20 | 0.38 | 0.42 | 0.49 | 0.00 |  |  |  |  |  |  |  |  |  |  |  |  |  |  |  |  |  |  |  |  |  |  |  |  |  |  |  |  |  |  |  |  |  |  |  |  |  |  |  |  |  |
| **H15** | 0.42 | 0.45 | 0.42 | 0.40 | 0.44 | 0.25 | 0.46 | 0.45 | 0.40 | 0.23 | 0.38 | 0.38 | 0.46 | 0.11 | 0.00 |  |  |  |  |  |  |  |  |  |  |  |  |  |  |  |  |  |  |  |  |  |  |  |  |  |  |  |  |  |  |  |  |  |  |  |  |  |  |  |  |
| **H16** | 0.33 | 0.36 | 0.42 | 0.45 | 0.48 | 0.42 | 0.46 | 0.49 | 0.47 | 0.46 | 0.27 | 0.51 | 0.36 | 0.42 | 0.42 | 0.00 |  |  |  |  |  |  |  |  |  |  |  |  |  |  |  |  |  |  |  |  |  |  |  |  |  |  |  |  |  |  |  |  |  |  |  |  |  |  |  |
| **H17** | 0.35 | 0.30 | 0.46 | 0.46 | 0.45 | 0.48 | 0.42 | 0.49 | 0.47 | 0.47 | 0.33 | 0.49 | 0.33 | 0.42 | 0.42 | 0.31 | 0.00 |  |  |  |  |  |  |  |  |  |  |  |  |  |  |  |  |  |  |  |  |  |  |  |  |  |  |  |  |  |  |  |  |  |  |  |  |  |  |
| **H18** | 0.52 | 0.55 | 0.52 | 0.51 | 0.62 | 0.55 | 0.54 | 0.32 | 0.51 | 0.52 | 0.56 | 0.58 | 0.60 | 0.53 | 0.54 | 0.57 | 0.57 | 0.00 |  |  |  |  |  |  |  |  |  |  |  |  |  |  |  |  |  |  |  |  |  |  |  |  |  |  |  |  |  |  |  |  |  |  |  |  |  |
| **H19** | 0.56 | 0.51 | 0.48 | 0.57 | 0.55 | 0.45 | 0.51 | 0.59 | 0.57 | 0.48 | 0.53 | 0.25 | 0.46 | 0.48 | 0.47 | 0.54 | 0.55 | 0.65 | 0.00 |  |  |  |  |  |  |  |  |  |  |  |  |  |  |  |  |  |  |  |  |  |  |  |  |  |  |  |  |  |  |  |  |  |  |  |  |
| **H20** | 0.35 | 0.41 | 0.42 | 0.49 | 0.49 | 0.42 | 0.48 | 0.53 | 0.52 | 0.51 | 0.30 | 0.42 | 0.28 | 0.49 | 0.47 | 0.30 | 0.40 | 0.59 | 0.46 | 0.00 |  |  |  |  |  |  |  |  |  |  |  |  |  |  |  |  |  |  |  |  |  |  |  |  |  |  |  |  |  |  |  |  |  |  |  |
| **H21** | 0.45 | 0.42 | 0.44 | 0.45 | 0.43 | 0.42 | 0.36 | 0.31 | 0.47 | 0.47 | 0.43 | 0.45 | 0.43 | 0.42 | 0.41 | 0.43 | 0.42 | 0.41 | 0.53 | 0.47 | 0.00 |  |  |  |  |  |  |  |  |  |  |  |  |  |  |  |  |  |  |  |  |  |  |  |  |  |  |  |  |  |  |  |  |  |  |
| **H22** | 0.47 | 0.42 | 0.35 | 0.41 | 0.36 | 0.47 | 0.29 | 0.45 | 0.38 | 0.44 | 0.41 | 0.50 | 0.45 | 0.41 | 0.42 | 0.42 | 0.42 | 0.53 | 0.53 | 0.50 | 0.34 | 0.00 |  |  |  |  |  |  |  |  |  |  |  |  |  |  |  |  |  |  |  |  |  |  |  |  |  |  |  |  |  |  |  |  |  |
| **L1** | 0.55 | 0.57 | 0.36 | 0.45 | 0.49 | 0.51 | 0.57 | 0.59 | 0.41 | 0.56 | 0.51 | 0.51 | 0.54 | 0.55 | 0.55 | 0.55 | 0.62 | 0.65 | 0.42 | 0.47 | 0.56 | 0.53 | 0.00 |  |  |  |  |  |  |  |  |  |  |  |  |  |  |  |  |  |  |  |  |  |  |  |  |  |  |  |  |  |  |  |  |
| **L2** | 0.54 | 0.57 | 0.37 | 0.43 | 0.42 | 0.45 | 0.53 | 0.58 | 0.40 | 0.54 | 0.48 | 0.53 | 0.51 | 0.51 | 0.51 | 0.53 | 0.58 | 0.62 | 0.48 | 0.46 | 0.53 | 0.51 | 0.21 | 0.00 |  |  |  |  |  |  |  |  |  |  |  |  |  |  |  |  |  |  |  |  |  |  |  |  |  |  |  |  |  |  |  |
| **L3** | 0.42 | 0.31 | 0.46 | 0.53 | 0.44 | 0.46 | 0.43 | 0.54 | 0.50 | 0.51 | 0.32 | 0.43 | 0.21 | 0.49 | 0.48 | 0.37 | 0.39 | 0.60 | 0.45 | 0.31 | 0.45 | 0.45 | 0.48 | 0.46 | 0.00 |  |  |  |  |  |  |  |  |  |  |  |  |  |  |  |  |  |  |  |  |  |  |  |  |  |  |  |  |  |  |
| **L4** | 0.29 | 0.27 | 0.44 | 0.45 | 0.51 | 0.49 | 0.44 | 0.46 | 0.46 | 0.44 | 0.36 | 0.45 | 0.37 | 0.47 | 0.45 | 0.35 | 0.32 | 0.53 | 0.53 | 0.42 | 0.43 | 0.44 | 0.58 | 0.57 | 0.34 | 0.00 |  |  |  |  |  |  |  |  |  |  |  |  |  |  |  |  |  |  |  |  |  |  |  |  |  |  |  |  |  |
| **L5** | 0.46 | 0.35 | 0.51 | 0.57 | 0.43 | 0.53 | 0.45 | 0.59 | 0.56 | 0.57 | 0.38 | 0.47 | 0.24 | 0.53 | 0.50 | 0.43 | 0.35 | 0.65 | 0.51 | 0.42 | 0.47 | 0.46 | 0.59 | 0.57 | 0.30 | 0.39 | 0.00 |  |  |  |  |  |  |  |  |  |  |  |  |  |  |  |  |  |  |  |  |  |  |  |  |  |  |  |  |
| **L6** | 0.31 | 0.31 | 0.43 | 0.45 | 0.40 | 0.42 | 0.45 | 0.51 | 0.49 | 0.47 | 0.30 | 0.44 | 0.25 | 0.44 | 0.42 | 0.32 | 0.30 | 0.57 | 0.51 | 0.33 | 0.40 | 0.44 | 0.55 | 0.51 | 0.27 | 0.32 | 0.27 | 0.00 |  |  |  |  |  |  |  |  |  |  |  |  |  |  |  |  |  |  |  |  |  |  |  |  |  |  |  |
| **L7** | 0.49 | 0.45 | 0.45 | 0.45 | 0.42 | 0.29 | 0.44 | 0.51 | 0.45 | 0.29 | 0.44 | 0.38 | 0.44 | 0.24 | 0.24 | 0.47 | 0.46 | 0.58 | 0.44 | 0.50 | 0.42 | 0.42 | 0.55 | 0.51 | 0.45 | 0.47 | 0.45 | 0.41 | 0.00 |  |  |  |  |  |  |  |  |  |  |  |  |  |  |  |  |  |  |  |  |  |  |  |  |  |  |
| **L8** | 0.44 | 0.46 | 0.27 | 0.29 | 0.33 | 0.41 | 0.49 | 0.47 | 0.32 | 0.44 | 0.40 | 0.49 | 0.46 | 0.39 | 0.38 | 0.44 | 0.45 | 0.54 | 0.56 | 0.47 | 0.41 | 0.38 | 0.44 | 0.37 | 0.47 | 0.45 | 0.50 | 0.42 | 0.42 | 0.00 |  |  |  |  |  |  |  |  |  |  |  |  |  |  |  |  |  |  |  |  |  |  |  |  |  |
| **L9** | 0.49 | 0.45 | 0.29 | 0.33 | 0.38 | 0.45 | 0.47 | 0.50 | 0.28 | 0.47 | 0.48 | 0.44 | 0.47 | 0.45 | 0.45 | 0.50 | 0.48 | 0.56 | 0.47 | 0.49 | 0.46 | 0.42 | 0.32 | 0.34 | 0.43 | 0.46 | 0.48 | 0.44 | 0.42 | 0.33 | 0.00 |  |  |  |  |  |  |  |  |  |  |  |  |  |  |  |  |  |  |  |  |  |  |  |  |
| **I1** | 0.42 | 0.47 | 0.39 | 0.41 | 0.42 | 0.23 | 0.44 | 0.49 | 0.42 | 0.21 | 0.38 | 0.39 | 0.45 | 0.18 | 0.18 | 0.41 | 0.46 | 0.55 | 0.45 | 0.45 | 0.42 | 0.39 | 0.54 | 0.50 | 0.46 | 0.45 | 0.50 | 0.42 | 0.21 | 0.40 | 0.47 | 0.00 |  |  |  |  |  |  |  |  |  |  |  |  |  |  |  |  |  |  |  |  |  |  |  |
| **I2** | 0.53 | 0.50 | 0.43 | 0.46 | 0.55 | 0.57 | 0.45 | 0.48 | 0.46 | 0.51 | 0.54 | 0.55 | 0.57 | 0.54 | 0.56 | 0.57 | 0.55 | 0.38 | 0.59 | 0.58 | 0.51 | 0.47 | 0.58 | 0.57 | 0.57 | 0.54 | 0.62 | 0.57 | 0.56 | 0.50 | 0.50 | 0.54 | 0.00 |  |  |  |  |  |  |  |  |  |  |  |  |  |  |  |  |  |  |  |  |  |  |
| **I3** | 0.53 | 0.54 | 0.50 | 0.49 | 0.58 | 0.53 | 0.50 | 0.39 | 0.49 | 0.51 | 0.53 | 0.56 | 0.56 | 0.51 | 0.52 | 0.57 | 0.57 | 0.25 | 0.62 | 0.57 | 0.42 | 0.51 | 0.59 | 0.59 | 0.58 | 0.56 | 0.60 | 0.54 | 0.53 | 0.53 | 0.53 | 0.53 | 0.39 | 0.00 |  |  |  |  |  |  |  |  |  |  |  |  |  |  |  |  |  |  |  |  |  |
| **I4** | 0.45 | 0.42 | 0.43 | 0.43 | 0.53 | 0.49 | 0.30 | 0.43 | 0.44 | 0.44 | 0.47 | 0.54 | 0.53 | 0.46 | 0.49 | 0.47 | 0.49 | 0.49 | 0.58 | 0.54 | 0.40 | 0.36 | 0.59 | 0.57 | 0.53 | 0.45 | 0.56 | 0.50 | 0.50 | 0.50 | 0.51 | 0.47 | 0.44 | 0.47 | 0.00 |  |  |  |  |  |  |  |  |  |  |  |  |  |  |  |  |  |  |  |  |
| **I5** | 0.40 | 0.44 | 0.40 | 0.38 | 0.53 | 0.47 | 0.40 | 0.34 | 0.41 | 0.38 | 0.47 | 0.54 | 0.54 | 0.42 | 0.45 | 0.47 | 0.47 | 0.41 | 0.60 | 0.54 | 0.39 | 0.37 | 0.62 | 0.59 | 0.54 | 0.42 | 0.57 | 0.48 | 0.49 | 0.46 | 0.50 | 0.43 | 0.43 | 0.45 | 0.24 | 0.00 |  |  |  |  |  |  |  |  |  |  |  |  |  |  |  |  |  |  |  |
| **I6** | 0.46 | 0.42 | 0.45 | 0.45 | 0.53 | 0.51 | 0.29 | 0.45 | 0.45 | 0.45 | 0.48 | 0.54 | 0.53 | 0.47 | 0.50 | 0.49 | 0.50 | 0.51 | 0.58 | 0.55 | 0.41 | 0.37 | 0.59 | 0.58 | 0.53 | 0.46 | 0.57 | 0.51 | 0.51 | 0.51 | 0.51 | 0.48 | 0.45 | 0.48 | 0.08 | 0.28 | 0.00 |  |  |  |  |  |  |  |  |  |  |  |  |  |  |  |  |  |  |
| **I7** | 0.49 | 0.49 | 0.48 | 0.49 | 0.55 | 0.54 | 0.41 | 0.45 | 0.49 | 0.49 | 0.51 | 0.54 | 0.54 | 0.51 | 0.51 | 0.51 | 0.53 | 0.33 | 0.61 | 0.56 | 0.44 | 0.42 | 0.63 | 0.62 | 0.55 | 0.48 | 0.58 | 0.53 | 0.54 | 0.54 | 0.55 | 0.50 | 0.34 | 0.32 | 0.36 | 0.37 | 0.37 | 0.00 |  |  |  |  |  |  |  |  |  |  |  |  |  |  |  |  |  |
| **I8** | 0.38 | 0.35 | 0.42 | 0.49 | 0.58 | 0.51 | 0.52 | 0.49 | 0.47 | 0.48 | 0.33 | 0.48 | 0.42 | 0.49 | 0.50 | 0.31 | 0.40 | 0.57 | 0.48 | 0.35 | 0.51 | 0.47 | 0.51 | 0.55 | 0.38 | 0.36 | 0.49 | 0.42 | 0.54 | 0.51 | 0.48 | 0.49 | 0.54 | 0.58 | 0.52 | 0.49 | 0.54 | 0.54 | 0.00 |  |  |  |  |  |  |  |  |  |  |  |  |  |  |  |  |
| **I9** | 0.45 | 0.47 | 0.45 | 0.42 | 0.56 | 0.48 | 0.47 | 0.08 | 0.42 | 0.43 | 0.48 | 0.51 | 0.54 | 0.44 | 0.45 | 0.49 | 0.48 | 0.31 | 0.59 | 0.53 | 0.30 | 0.45 | 0.59 | 0.57 | 0.54 | 0.46 | 0.59 | 0.51 | 0.51 | 0.46 | 0.49 | 0.49 | 0.48 | 0.38 | 0.43 | 0.34 | 0.45 | 0.45 | 0.49 | 0.00 |  |  |  |  |  |  |  |  |  |  |  |  |  |  |  |
| **I10** | 0.42 | 0.47 | 0.42 | 0.40 | 0.57 | 0.49 | 0.47 | 0.38 | 0.42 | 0.40 | 0.45 | 0.54 | 0.55 | 0.42 | 0.45 | 0.50 | 0.51 | 0.34 | 0.60 | 0.54 | 0.43 | 0.43 | 0.58 | 0.57 | 0.54 | 0.47 | 0.60 | 0.50 | 0.50 | 0.45 | 0.50 | 0.46 | 0.34 | 0.36 | 0.38 | 0.31 | 0.40 | 0.33 | 0.50 | 0.38 | 0.00 |  |  |  |  |  |  |  |  |  |  |  |  |  |  |
| **I11** | 0.57 | 0.47 | 0.47 | 0.56 | 0.55 | 0.42 | 0.50 | 0.55 | 0.51 | 0.43 | 0.51 | 0.34 | 0.49 | 0.42 | 0.42 | 0.51 | 0.54 | 0.61 | 0.30 | 0.50 | 0.51 | 0.47 | 0.47 | 0.52 | 0.42 | 0.47 | 0.52 | 0.53 | 0.40 | 0.52 | 0.43 | 0.41 | 0.57 | 0.61 | 0.55 | 0.54 | 0.55 | 0.57 | 0.42 | 0.55 | 0.56 | 0.00 |  |  |  |  |  |  |  |  |  |  |  |  |  |
| **I12** | 0.56 | 0.55 | 0.38 | 0.46 | 0.48 | 0.51 | 0.56 | 0.57 | 0.41 | 0.57 | 0.51 | 0.48 | 0.50 | 0.55 | 0.53 | 0.56 | 0.59 | 0.62 | 0.44 | 0.48 | 0.54 | 0.54 | 0.21 | 0.28 | 0.48 | 0.58 | 0.56 | 0.54 | 0.55 | 0.41 | 0.32 | 0.56 | 0.59 | 0.57 | 0.60 | 0.62 | 0.61 | 0.62 | 0.54 | 0.55 | 0.59 | 0.49 | 0.00 |  |  |  |  |  |  |  |  |  |  |  |  |
| **I13** | 0.51 | 0.53 | 0.49 | 0.49 | 0.51 | 0.34 | 0.47 | 0.43 | 0.49 | 0.38 | 0.47 | 0.40 | 0.51 | 0.35 | 0.35 | 0.51 | 0.50 | 0.51 | 0.42 | 0.49 | 0.42 | 0.47 | 0.51 | 0.48 | 0.51 | 0.51 | 0.56 | 0.49 | 0.40 | 0.47 | 0.51 | 0.37 | 0.54 | 0.51 | 0.51 | 0.47 | 0.53 | 0.53 | 0.53 | 0.43 | 0.47 | 0.46 | 0.51 | 0.00 |  |  |  |  |  |  |  |  |  |  |  |
| **I14** | 0.58 | 0.49 | 0.44 | 0.49 | 0.45 | 0.57 | 0.45 | 0.55 | 0.47 | 0.58 | 0.57 | 0.49 | 0.51 | 0.58 | 0.57 | 0.56 | 0.50 | 0.60 | 0.48 | 0.57 | 0.45 | 0.46 | 0.45 | 0.46 | 0.50 | 0.48 | 0.51 | 0.54 | 0.53 | 0.47 | 0.37 | 0.57 | 0.57 | 0.60 | 0.55 | 0.59 | 0.54 | 0.59 | 0.57 | 0.54 | 0.62 | 0.50 | 0.45 | 0.56 | 0.00 |  |  |  |  |  |  |  |  |  |  |
| **I15** | 0.54 | 0.51 | 0.41 | 0.49 | 0.42 | 0.47 | 0.47 | 0.53 | 0.48 | 0.54 | 0.49 | 0.44 | 0.45 | 0.52 | 0.52 | 0.50 | 0.53 | 0.59 | 0.38 | 0.43 | 0.45 | 0.49 | 0.35 | 0.34 | 0.39 | 0.51 | 0.48 | 0.45 | 0.47 | 0.45 | 0.36 | 0.49 | 0.54 | 0.55 | 0.56 | 0.57 | 0.57 | 0.60 | 0.48 | 0.53 | 0.57 | 0.44 | 0.40 | 0.48 | 0.38 | 0.00 |  |  |  |  |  |  |  |  |  |
| **I16** | 0.50 | 0.53 | 0.45 | 0.51 | 0.50 | 0.30 | 0.51 | 0.53 | 0.52 | 0.41 | 0.45 | 0.34 | 0.45 | 0.38 | 0.37 | 0.47 | 0.55 | 0.59 | 0.31 | 0.38 | 0.47 | 0.51 | 0.43 | 0.42 | 0.45 | 0.54 | 0.55 | 0.47 | 0.40 | 0.49 | 0.50 | 0.34 | 0.60 | 0.57 | 0.55 | 0.55 | 0.57 | 0.57 | 0.50 | 0.53 | 0.55 | 0.40 | 0.44 | 0.31 | 0.56 | 0.42 | 0.00 |  |  |  |  |  |  |  |  |
| **W1** | 0.49 | 0.53 | 0.46 | 0.49 | 0.57 | 0.36 | 0.54 | 0.43 | 0.49 | 0.41 | 0.49 | 0.36 | 0.51 | 0.41 | 0.40 | 0.54 | 0.56 | 0.50 | 0.42 | 0.47 | 0.45 | 0.57 | 0.48 | 0.47 | 0.51 | 0.54 | 0.58 | 0.53 | 0.43 | 0.50 | 0.48 | 0.42 | 0.58 | 0.50 | 0.54 | 0.54 | 0.55 | 0.57 | 0.54 | 0.42 | 0.53 | 0.44 | 0.47 | 0.40 | 0.53 | 0.47 | 0.37 | 0.00 |  |  |  |  |  |  |  |
| **W2** | 0.60 | 0.60 | 0.60 | 0.59 | 0.68 | 0.49 | 0.67 | 0.46 | 0.62 | 0.53 | 0.63 | 0.48 | 0.62 | 0.52 | 0.49 | 0.63 | 0.63 | 0.53 | 0.58 | 0.60 | 0.50 | 0.67 | 0.66 | 0.64 | 0.64 | 0.60 | 0.68 | 0.62 | 0.54 | 0.58 | 0.60 | 0.54 | 0.67 | 0.58 | 0.65 | 0.60 | 0.67 | 0.64 | 0.64 | 0.45 | 0.62 | 0.57 | 0.60 | 0.51 | 0.63 | 0.62 | 0.51 | 0.38 | 0.00 |  |  |  |  |  |  |
| **W3** | 0.62 | 0.62 | 0.62 | 0.60 | 0.69 | 0.51 | 0.67 | 0.44 | 0.62 | 0.55 | 0.64 | 0.51 | 0.64 | 0.54 | 0.52 | 0.65 | 0.65 | 0.51 | 0.60 | 0.63 | 0.51 | 0.68 | 0.67 | 0.65 | 0.65 | 0.61 | 0.69 | 0.64 | 0.57 | 0.60 | 0.61 | 0.57 | 0.67 | 0.58 | 0.66 | 0.62 | 0.67 | 0.66 | 0.66 | 0.42 | 0.63 | 0.58 | 0.62 | 0.51 | 0.64 | 0.63 | 0.54 | 0.38 | 0.20 | 0.00 |  |  |  |  |  |
| **W4** | 0.59 | 0.60 | 0.59 | 0.58 | 0.67 | 0.49 | 0.65 | 0.44 | 0.59 | 0.51 | 0.62 | 0.47 | 0.61 | 0.51 | 0.49 | 0.63 | 0.63 | 0.51 | 0.57 | 0.60 | 0.51 | 0.67 | 0.65 | 0.63 | 0.63 | 0.58 | 0.68 | 0.62 | 0.54 | 0.58 | 0.58 | 0.54 | 0.66 | 0.57 | 0.65 | 0.60 | 0.66 | 0.64 | 0.64 | 0.42 | 0.61 | 0.55 | 0.59 | 0.49 | 0.62 | 0.60 | 0.51 | 0.36 | 0.18 | 0.14 | 0.00 |  |  |  |  |
| **W5** | 0.55 | 0.59 | 0.57 | 0.57 | 0.64 | 0.47 | 0.63 | 0.45 | 0.60 | 0.51 | 0.60 | 0.45 | 0.59 | 0.52 | 0.49 | 0.59 | 0.63 | 0.52 | 0.55 | 0.57 | 0.49 | 0.64 | 0.65 | 0.63 | 0.61 | 0.55 | 0.66 | 0.58 | 0.54 | 0.58 | 0.61 | 0.51 | 0.67 | 0.56 | 0.61 | 0.56 | 0.63 | 0.60 | 0.62 | 0.45 | 0.59 | 0.56 | 0.62 | 0.48 | 0.62 | 0.58 | 0.47 | 0.37 | 0.25 | 0.25 | 0.21 | 0.00 |  |  |  |
| **W6** | 0.57 | 0.59 | 0.57 | 0.57 | 0.65 | 0.48 | 0.64 | 0.44 | 0.61 | 0.51 | 0.61 | 0.45 | 0.59 | 0.53 | 0.51 | 0.62 | 0.64 | 0.51 | 0.55 | 0.58 | 0.49 | 0.65 | 0.65 | 0.63 | 0.61 | 0.57 | 0.66 | 0.59 | 0.54 | 0.57 | 0.61 | 0.52 | 0.66 | 0.55 | 0.62 | 0.57 | 0.64 | 0.62 | 0.63 | 0.44 | 0.59 | 0.56 | 0.62 | 0.47 | 0.62 | 0.58 | 0.49 | 0.35 | 0.25 | 0.23 | 0.21 | 0.11 | 0.00 |  |  |
| **W7** | 0.59 | 0.60 | 0.59 | 0.58 | 0.67 | 0.47 | 0.66 | 0.45 | 0.60 | 0.53 | 0.62 | 0.47 | 0.61 | 0.51 | 0.49 | 0.62 | 0.63 | 0.53 | 0.57 | 0.60 | 0.49 | 0.67 | 0.65 | 0.63 | 0.63 | 0.58 | 0.67 | 0.61 | 0.54 | 0.57 | 0.58 | 0.54 | 0.68 | 0.57 | 0.65 | 0.60 | 0.66 | 0.64 | 0.64 | 0.44 | 0.61 | 0.55 | 0.59 | 0.49 | 0.62 | 0.60 | 0.51 | 0.36 | 0.18 | 0.18 | 0.11 | 0.18 | 0.18 | 0.00 |  |
| **W8** | 0.55 | 0.57 | 0.55 | 0.55 | 0.64 | 0.51 | 0.62 | 0.40 | 0.59 | 0.54 | 0.59 | 0.49 | 0.58 | 0.55 | 0.54 | 0.60 | 0.62 | 0.48 | 0.58 | 0.57 | 0.47 | 0.63 | 0.64 | 0.62 | 0.59 | 0.55 | 0.64 | 0.58 | 0.57 | 0.56 | 0.59 | 0.55 | 0.63 | 0.54 | 0.60 | 0.55 | 0.62 | 0.60 | 0.61 | 0.40 | 0.58 | 0.59 | 0.60 | 0.51 | 0.60 | 0.57 | 0.52 | 0.39 | 0.29 | 0.24 | 0.25 | 0.21 | 0.18 | 0.25 | 0.00 |

Note. Germplasm codes are shown in Table S1.


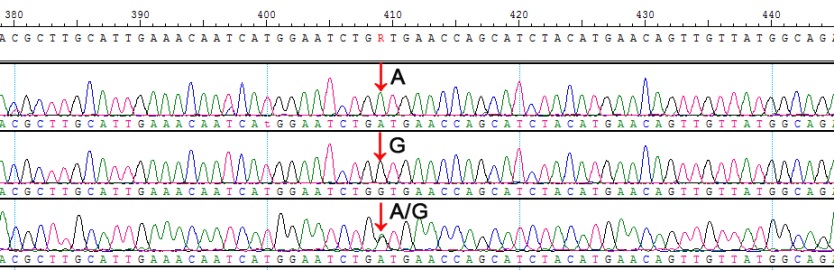


A


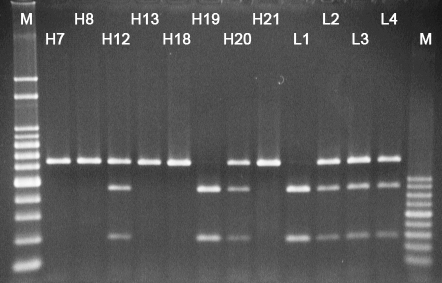


B

**Additional file** **Figure S1.** A. Partial nucleotide sequences of three cultivars amplified with G01 primer set, and arrow points indicated SNP sites. B. G01 CAPS marker designed from SNP sites of G01 primer set.

| 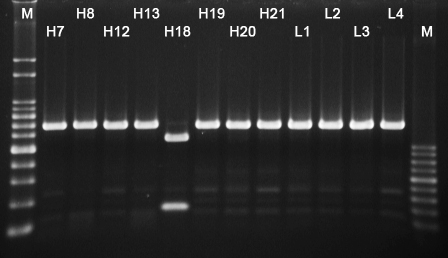 | 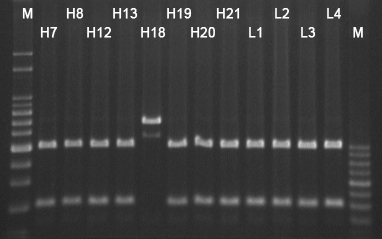 |
| --- | --- |
| C01 | C02 |
| 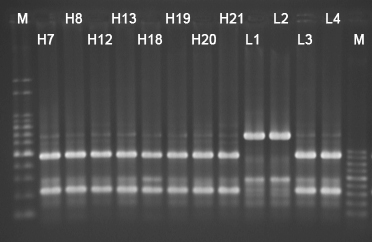 | 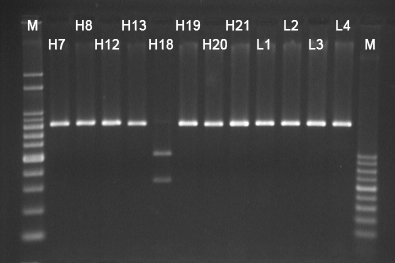 |
| C03 | M01 |
| 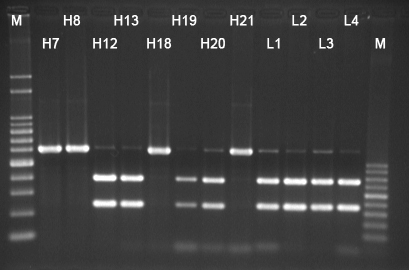 | 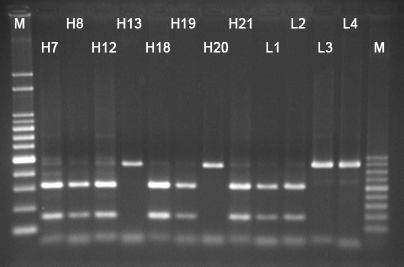 |
| M02 | M03 |
| 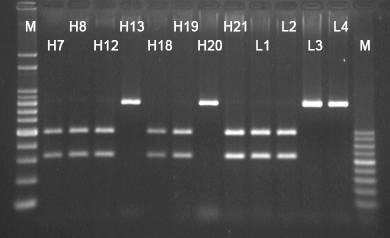 | 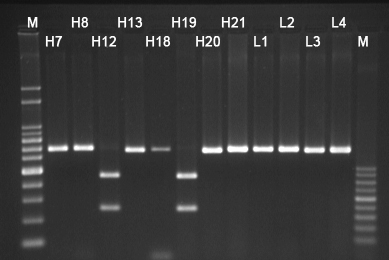 |
| M04 | M05 |
| **Additional file** **Figure S2.** The cleaved fragment patterns of each STS and CAPS marker for 12 prevailing tea cultivars in Taiwan. | |
| 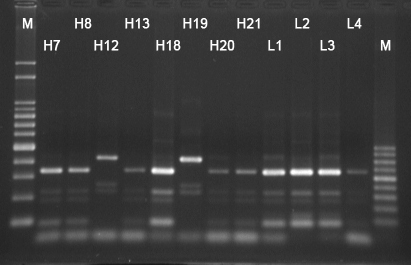 | 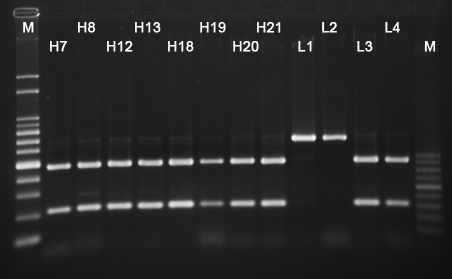 |
| M06 | M07 |
| 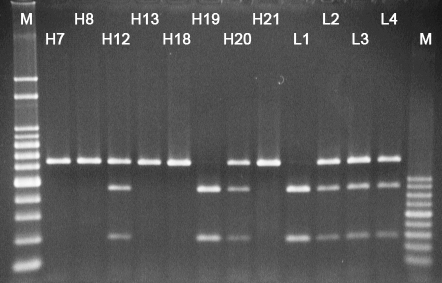 | 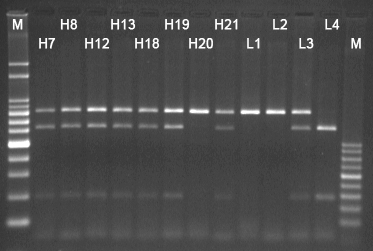 |
| G01 | G02 |
| 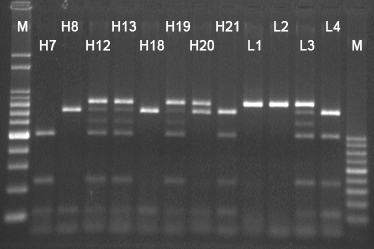 | 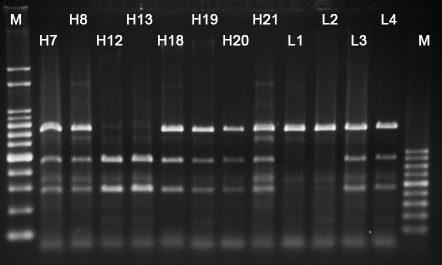 |
| G03 | G04 |
| 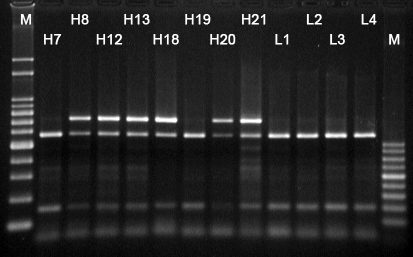 | 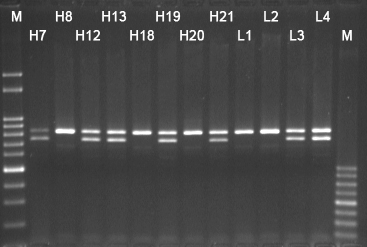 |
| G05 | G06 |
| **Additional file** **Figure S2.** Continued | |
| 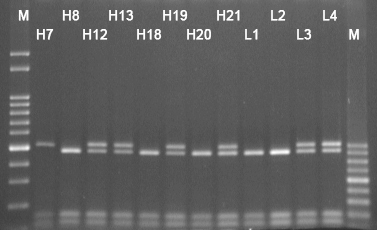 | 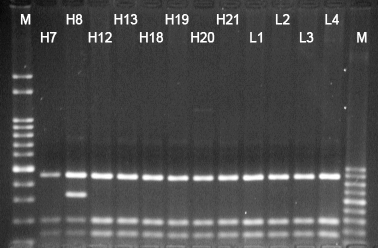 |
| G07 | G08 |
| 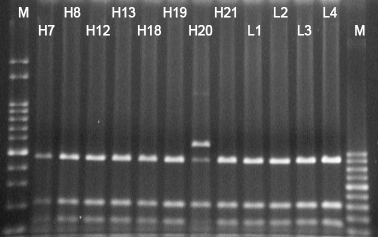 | 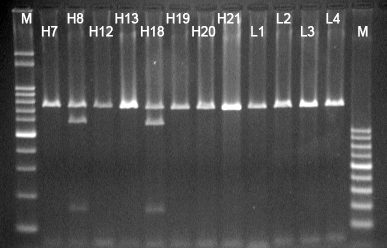 |
| G09 | G10 |
| 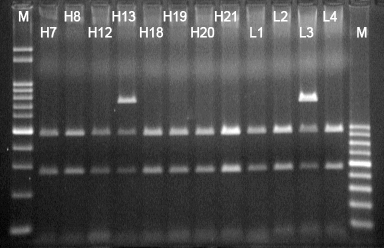 | 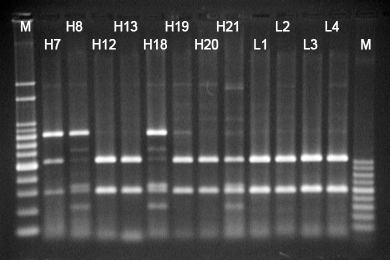 |
| G11 | G12 |
| 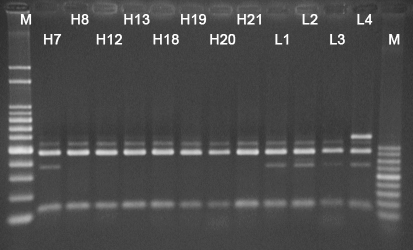 | 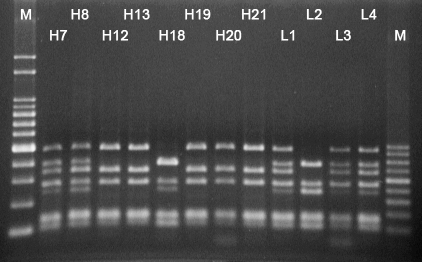 |
| G13 | G14 |
| **Additional file** **Figure S2.** Continued | |
| 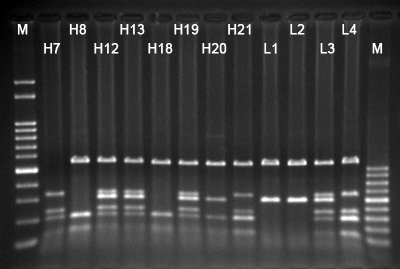 | 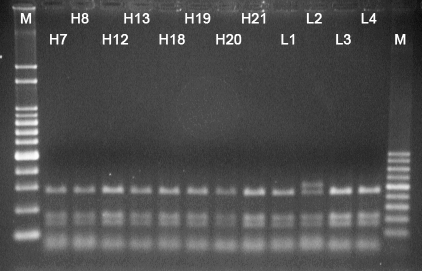 |
| G15 | G16 |
| 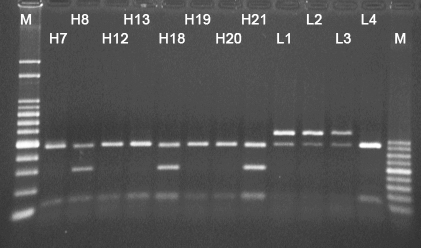 | 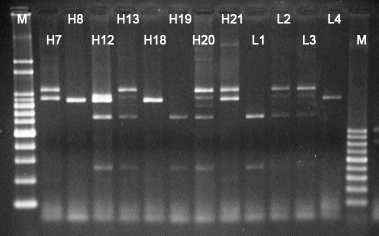 |
| G17 | G18 |
| 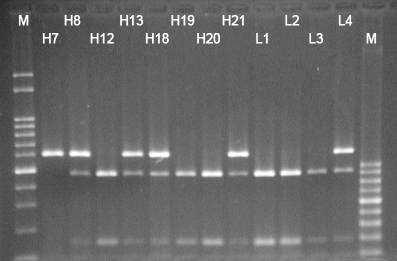 | 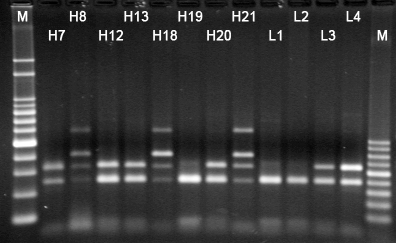 |
| G19 | G20 |
| 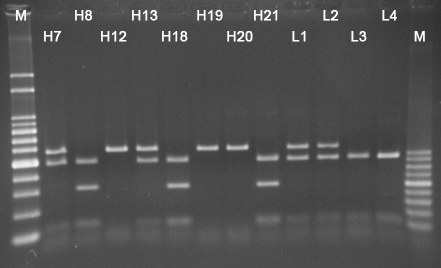 | 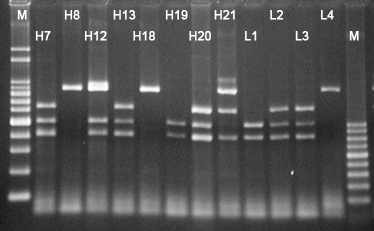 |
| G21 | G22 |
| **Additional file** **Figure S2.** Continued | |
| 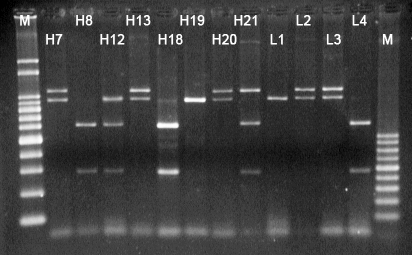 | 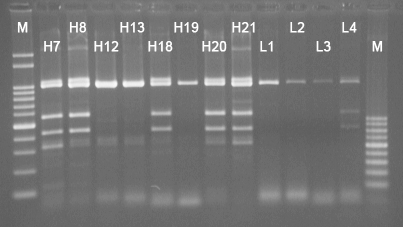 |
| G23 | G24 |
| 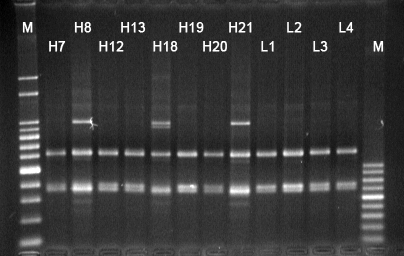 | 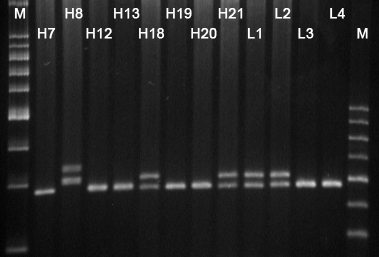 |
| G25 | G26 |
| 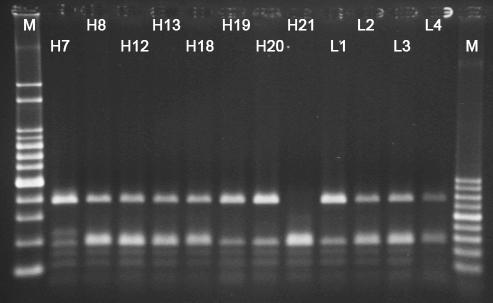 | 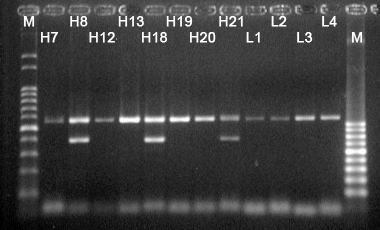 |
| G27 | PAL |
| 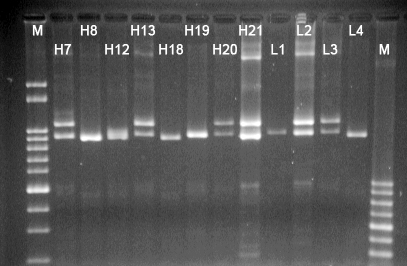 |  |
| F3H |  |
| **Additional file** **Figure S2.** Continued | |
