# Supplementary figures and images for: Development of STS and CAPS markers for variety identification and genetic diversity analysis of tea germplasm in Taiwan
Source: Bot Stud. 2014 Feb 1;55:12. doi: 10.1186/1999-3110-55-12 (PMC5430312; doi:10.1186/1999-3110-55-12)

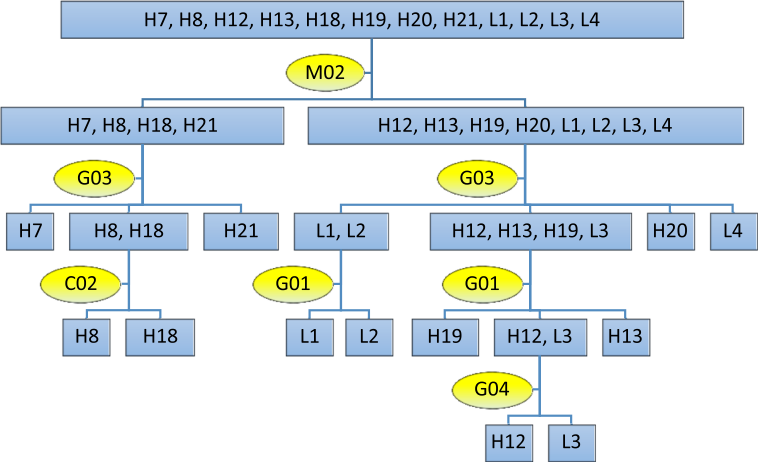

Supplement: Supplementary file 2 — Authors’ original file for figure 1 [file 40529_2013_67_MOESM2_ESM.pdf]

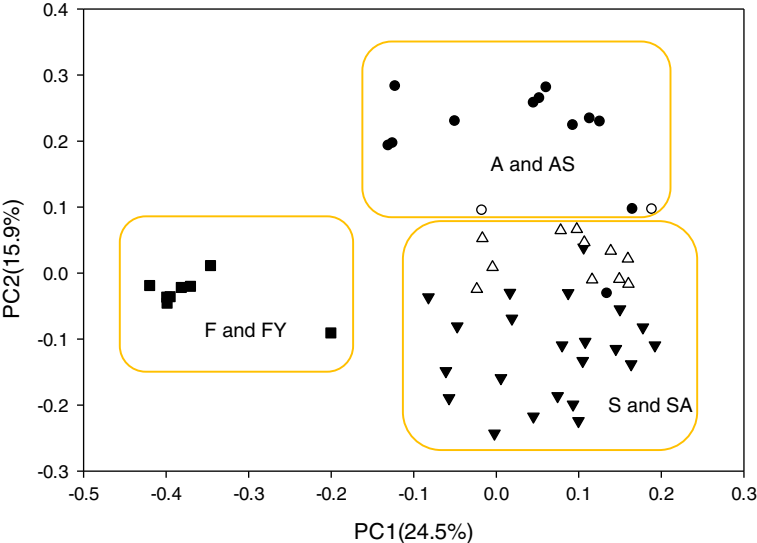

Supplement: Supplementary file 3 — Authors’ original file for figure 2 [file 40529_2013_67_MOESM3_ESM.pdf]

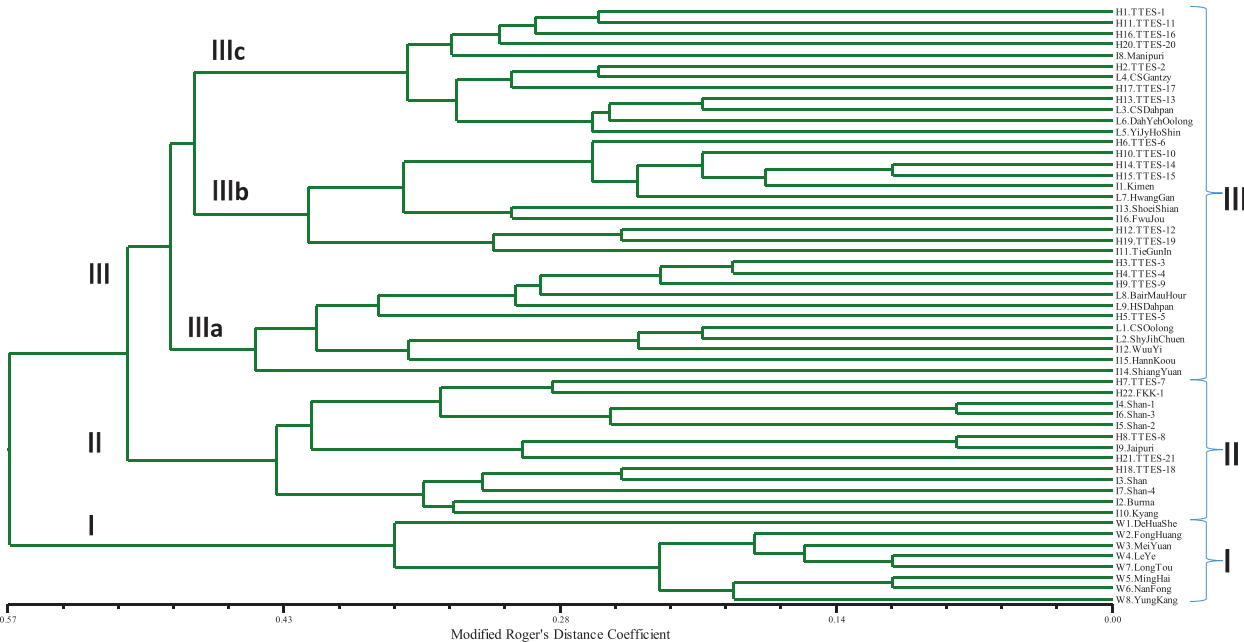

Supplement: Supplementary file 4 — Authors’ original file for figure 3 [file 40529_2013_67_MOESM4_ESM.pdf]
